# Musculoskeletal Ultrasound and Protein-Based Hydrogels: Novel Approaches for the Diagnosis and Treatment of Sports Injuries

**DOI:** 10.3390/polym18111272

**Published:** 2026-05-22

**Authors:** Hongchao Zhang, Xing Gao, Yihao Yan

**Affiliations:** Graduate School, School of Kinesiology and Health, Harbin Sport University, Harbin 150008, China; a13351455315@163.com (H.Z.); yyh18154365585@163.com (Y.Y.)

**Keywords:** musculoskeletal ultrasound, protein-based hydrogels, injury diagnosis, sports rehabilitation

## Abstract

Sports-related injuries involving muscle, tendon, cartilage, ligament, and bone remain challenging because of their heterogeneous mechanisms, high functional demands, and need for timely diagnosis, targeted repair, and rehabilitation monitoring. Musculoskeletal ultrasound (MSKUS) provides real-time, portable, radiation-free, and repeatable evaluation of injured tissues, including lesion location, structural continuity, vascular response, stiffness changes, and healing progression. Protein-based hydrogels, owing to their biocompatibility, tunable mechanics, extracellular matrix-like microenvironments, and capacity for localized bioactive delivery, offer promising platforms for tissue repair and functional recovery. This review summarizes major sports injuries, discusses the diagnostic and monitoring value of MSKUS, and analyzes protein-based hydrogels according to tissue-specific repair requirements. Particular attention is given to the connection between imaging assessment and hydrogel therapy, including ultrasound-guided delivery, lesion localization, post-treatment monitoring of hydrogel retention or degradation, and evaluation of therapeutic response. By linking MSKUS-based lesion assessment with hydrogel selection, image-guided delivery, post-intervention monitoring, and rehabilitation-oriented decision-making, this review outlines a coordinated framework for precise diagnosis and localized biomaterial-assisted repair in sports injury management.

## 1. Introduction

With increasing health awareness and the growing popularity of competitive sports, exercise has become an important means of improving physical fitness and quality of life [1]. However, sports injuries represent the most common health threat in this process and exhibit distinct characteristics compared with trauma or degenerative diseases [2]. These injuries often occur in high-intensity, complex, and repetitive athletic environments. In such settings, acute mechanical impact may be compounded by chronic overload, ultimately leading to structural damage and functional impairment of muscles, tendons, cartilage, and ligaments [3]. Epidemiological data indicate a continuous rise in the incidence of sports injuries, which not only affects daily activities and athletic performance but also imposes greater demands on the diagnostic and therapeutic systems of sports medicine [4,5].

Although conventional diagnostic and therapeutic approaches are widely applied, they face significant limitations in the unique context of sports injuries [6]. Imaging modalities such as MRI, CT, and X-ray provide valuable structural information, but they may be limited in bedside availability, dynamic functional assessment, repeated follow-up, or radiation exposure depending on the modality [7,8]. Therapeutically, surgery, pharmacological intervention, and rehabilitation training provide certain benefits. However, these approaches often remain limited in repair speed, tissue regeneration quality, and functional restoration, making it difficult to meet athletes’ urgent need for “rapid return to play” [9,10,11]. Consequently, sports medicine requires the development of novel diagnostic and therapeutic strategies tailored to athletic scenarios.

Against this backdrop, musculoskeletal ultrasound (MSKUS) has emerged as a promising imaging tool due to its radiation-free nature, operational convenience, and capability for real-time dynamic imaging. MSKUS not only captures the dynamic changes of tendons, ligaments, and muscles during motion execution but also enables rapid bedside assessment of injured tissues and monitoring of the rehabilitation process, making it particularly suitable for populations requiring frequent follow-up and immediate feedback [12]. At the same time, protein-based hydrogels, with their excellent biocompatibility, tunable mechanical properties, and biomimetic reparative features, show significant potential in tissue regeneration, inflammation modulation, and functional restoration after sports injuries [13,14]. They can serve as delivery platforms for drugs and growth factors while providing a three-dimensional extracellular matrix (ECM)-like environment, thereby creating favorable conditions for tissue repair and regeneration [15].

Accordingly, this review discusses sports injury management as a continuous process involving injury evaluation, targeted intervention, and rehabilitation-oriented follow-up. First, the major types of sports injuries and their current clinical challenges are summarized to clarify the need for rapid diagnosis and tissue-specific repair. The role of MSKUS is then discussed, with emphasis on dynamic diagnosis, lesion localization, severity assessment, interventional guidance, and follow-up evaluation. Building on this imaging-based assessment, the review further examines how MSKUS-derived information, including lesion location, tissue depth, structural disruption, vascular response, effusion, and stiffness, may help inform hydrogel selection, delivery route, and post-treatment monitoring strategy. Finally, protein-based hydrogels are analyzed according to their repair-oriented functions in different injured tissues, including inflammation regulation, extracellular matrix remodeling, controlled bioactive delivery, mechanical support, and tissue-specific regeneration. By connecting dynamic imaging evaluation with localized biomaterial-assisted repair, this review aims to provide a practical and integrated perspective for precise diagnosis, image-guided intervention, therapeutic monitoring, and rehabilitation-oriented management of sports injuries.

## 2. Literature Search Strategy and Source Selection

This review was designed as a narrative review informed by a structured literature search and source selection process. Relevant studies were identified from PubMed/MEDLINE, Web of Science, Scopus, and Google Scholar. The search covered publications from 2014 to 2025, with priority given to studies published within the last five years when available. Earlier studies were also included when they provided important methodological, clinical, or mechanistic information relevant to sports injury diagnosis, musculoskeletal ultrasound, or protein-based hydrogel-mediated tissue repair.

The search strategy was organized around three major themes: sports-related musculoskeletal injuries, musculoskeletal ultrasound, and protein-based hydrogels. The main search terms included “sports injury”, “musculoskeletal injury”, “muscle injury”, “tendon injury”, “cartilage injury”, “ligament injury”, “fracture”, “sports rehabilitation”, “musculoskeletal ultrasound”, “ultrasound imaging”, “dynamic ultrasound”, “shear wave elastography”, “ultrasound-guided intervention”, “protein-based hydrogel”, “natural protein hydrogel”, “GelMA”, “gelatin hydrogel”, “collagen hydrogel”, “silk fibroin hydrogel”, “fibrin hydrogel”, “tissue repair”, “regenerative medicine”, and “biomaterial-assisted repair”. Boolean operators, including AND and OR, were used to combine diagnostic, therapeutic, and biomaterial-related terms.

The source selection process was performed in three steps. First, titles and abstracts were screened to identify studies related to sports injury diagnosis, MSKUS-based evaluation, hydrogel-assisted tissue repair, or sports rehabilitation. Second, potentially relevant articles were assessed through full-text reading to determine whether they provided sufficient methodological details, experimental evidence, clinical relevance, or mechanistic interpretation. Third, the selected literature was organized according to the structure of this review, including sports injury characteristics, MSKUS-based diagnosis and monitoring, protein-based hydrogel-mediated repair, and translational perspectives.

Studies were included if they met at least one of the following criteria: they reported or reviewed sports-related musculoskeletal injuries involving muscle, tendon, ligament, cartilage, bone, or tendon-bone interfaces; they evaluated the diagnostic, dynamic monitoring, or interventional value of MSKUS or ultrasound-based techniques; they described protein-based hydrogel systems for musculoskeletal tissue repair or regenerative medicine; or they provided quantitative or mechanistic evidence relevant to biomaterial-assisted repair, tissue regeneration, or rehabilitation monitoring. Studies were excluded if they were unrelated to musculoskeletal injury or tissue repair, focused on non-protein-based hydrogels without relevance to the topic, lacked accessible full text, were conference abstracts without sufficient methodological information, or were duplicate publications.

Because this review is narrative in scope rather than a systematic review or meta-analysis, no formal quantitative synthesis or risk-of-bias assessment was conducted. Instead, the included literature was qualitatively analyzed to construct an integrated framework linking sports injury diagnosis, MSKUS-based evaluation, protein-based hydrogel therapy, and rehabilitation follow-up.

## 3. Major Types of Sports Injuries and Current Therapeutic Approaches

Although exercise promotes health, it is inevitably associated with an inherent risk of injury [16]. Among them, muscle injuries (e.g., strains, contusions, and tears), tendon injuries (including tendinitis, tendinopathy, and ruptures), fractures (particularly stress fractures and acute traumatic fractures), and cartilage injuries (degenerative or traumatic articular cartilage lesions) represent the most common and impactful categories. These four types of injuries differ significantly in their etiologies, susceptible populations, typical clinical manifestations, and therapeutic pathways (Table 1).

### 3.1. Cartilage Injuries

Articular cartilage is a critical component of the musculoskeletal system that absorbs impact, distributes load, reduces friction, and maintains smooth joint motion [44]. However, because cartilage lacks blood vessels and innervation, its intrinsic self-repair capacity is extremely limited [45]. Once damaged, cartilage injuries can profoundly compromise both athletic performance and long-term joint health. These injuries are particularly common in sports requiring high-intensity impact or repetitive joint loading. For instance, abrupt stops, pivots, and collisions in contact sports such as basketball and soccer can lead to cartilage contusions or delamination in the knee joint. Endurance activities, including long-distance running and marathon training, may induce cartilage degeneration through chronic wear. High-load disciplines, such as weightlifting and gymnastics, may further cause cartilage fissures or focal defects [46].

Clinically, cartilage injuries manifest as joint pain, swelling, stiffness, and restricted mobility, with some patients experiencing locking, crepitus, or abnormal gait [47]. As damage progresses, muscle atrophy, strength loss, and impaired motor coordination may occur, eventually advancing to osteoarthritis, which severely impacts both athletic careers and quality of life.

From a pathophysiological perspective, cartilage injury is driven by both mechanical overload and biological degeneration. Acute trauma typically disrupts the extracellular matrix, causing collagen fiber rupture and chondrocyte necrosis [48]. Chronic wear, in contrast, is closely associated with the activation of pro-inflammatory cytokines, overexpression of matrix metalloproteinases (MMPs), metabolic imbalance of chondrocytes, and progressive extracellular matrix degradation [49]. Because chondrocytes exhibit poor regenerative capacity, the repair process often results in fibrocartilage with inferior biomechanical properties, failing to restore the buffering and lubricating functions of native hyaline cartilage [50].

Current treatment options include conservative interventions (e.g., hyaluronic acid or platelet-rich plasma [PRP] injections), surgical procedures (e.g., microfracture, autologous chondrocyte implantation, or allogeneic transplantation), and regenerative medicine approaches (e.g., stem cell–scaffold composites) [51]. Nevertheless, these strategies remain limited. Conservative therapies often provide only temporary symptom relief, surgically repaired tissue is prone to degeneration, and regenerative approaches still face challenges such as poor cell survival, limited scaffold mechanical integrity, and low translational efficiency.

Future directions lie in developing biomimetic and intelligent repair systems. On one hand, protein-based hydrogels can serve as scaffolds and delivery platforms for bioactive factors, offering a mechanical and biochemical microenvironment resembling native cartilage to promote hyaline-like regeneration. On the other, musculoskeletal ultrasound enables real-time evaluation and dynamic monitoring of cartilage damage, providing imaging-based guidance for personalized rehabilitation. Moreover, integrating multidisciplinary strategies—including sports rehabilitation, tissue engineering, and molecular regulation—together with AI-assisted rehabilitation assessment, may help overcome the limitations of fibrotic repair and achieve true functional restoration of articular cartilage.

### 3.2. Muscle Injuries

Skeletal muscle is the main effector tissue of the musculoskeletal system and is responsible for generating movement, maintaining posture, absorbing impact, and regulating metabolism [52]. Muscle injuries are extremely common in athletic settings, particularly in high-intensity, explosive, and repetitive activities [53]. For instance, abrupt stops or rapid accelerations in sprinting and soccer frequently cause hamstring and quadriceps strains; jumping and landing in basketball and volleyball often result in calf muscle tears; endurance activities such as long-distance running and triathlon predispose athletes to chronic overuse injuries and delayed-onset muscle soreness (DOMS); and extreme sports such as skateboarding and rock climbing may lead to volumetric muscle loss with substantial structural damage [54]. Clinically, acute manifestations include pain, swelling, tenderness, loss of strength, and restricted joint mobility, with severe cases presenting with visible deformity or hematoma formation. In the long term, recurrent injuries can lead to motor control dysfunction, compensatory movement patterns, and performance decline, substantially delaying or compromising an athlete’s return to play [55].

Pathophysiologically, muscle injuries generally result from the combined effects of mechanical overload and metabolic imbalance [56]. Acute injuries cause myofiber rupture and sarcolemma disruption, accompanied by calcium influx and myofibril disassembly; chronic injuries, however, are closely linked to oxidative stress, persistent inflammatory activation, and mitochondrial dysfunction. During repair, challenges often arise from replacement of myofibers by scar tissue, limited efficiency of satellite cell-mediated regeneration, and impaired reinnervation, all of which hinder functional recovery and increase the risk of recurrence [25].

Current therapeutic options include RICE (rest, ice, compression, and elevation) in combination with pharmacological treatments such as nonsteroidal anti-inflammatory drugs (NSAIDs), rehabilitation training, and surgical repair when necessary [57]. Regenerative medicine approaches—including stem cell transplantation, platelet-rich plasma (PRP) injections, and tissue engineering strategies—are also being increasingly explored [58]. However, each method has important limitations. Pharmacological treatments may disrupt the delicate balance between inflammation and repair; PRP preparation and application lack standardized protocols; stem cell therapy is constrained by low survival rates and insufficient myogenic efficiency; and surgery may result in functional deficits or secondary scarring [59,60,61,62,63].

Future directions emphasize the construction of more biomimetic and intelligent therapeutic systems. Protein-based hydrogels, for example, can act as delivery platforms for cells and growth factors, providing both biomechanical support and bioactive microenvironments conducive to muscle regeneration. In parallel, musculoskeletal ultrasound enables real-time monitoring for injury evaluation and dynamic tracking during rehabilitation. Furthermore, interdisciplinary strategies that combine sports rehabilitation, biomaterials, regenerative medicine, and personalized rehabilitation programs may help overcome current limitations and facilitate the transition from structural repair to functional muscle restoration.

### 3.3. Tendon Injuries

Tendons are essential load-bearing structures that connect muscles to bones, transmitting contractile forces to drive joint movement, stabilize joints, and absorb mechanical shocks [64]. In addition, Golgi tendon organs embedded within tendons detect changes in tensile load and provide feedback to the nervous system, thereby playing a key role in motor control and postural regulation [65]. Because tendons are exposed to high mechanical loads, they are highly vulnerable to sports-related injuries, particularly during high-intensity, repetitive, or explosive activities. For example, frequent jumping and landing in basketball and volleyball often lead to Achilles tendinopathy or partial ruptures; excessive forearm rotation and acceleration in racket sports such as tennis and badminton commonly result in lateral epicondylopathy (“tennis elbow”); endurance training, such as long-distance and marathon running, is strongly associated with patellar tendinopathy or tenosynovitis; and high-load sports such as weightlifting and gymnastics may trigger acute tendon ruptures [66,67,68,69].

Clinically, tendon injuries are characterized by localized pain and tenderness, reduced joint mobility, muscle stiffness, and impaired motor control. Patients often experience reduced muscle strength and endurance, and in severe cases even basic daily activities and athletic performance are significantly compromised. Recurrent or chronic tendon injuries not only cause functional disability but also induce compensatory movement patterns, leading to decreased performance and a heightened risk of re-injury [37,70,71].

Pathophysiologically, tendon injuries arise from a combination of mechanical overload, biological degeneration, and biomechanical imbalance [72,73,74]. Acute injuries often result in collagen fiber rupture and tenocyte necrosis, whereas chronic injuries are associated with persistent inflammatory activation, disrupted collagen synthesis–degradation balance, and a low-metabolic state of tenocytes. The repair process frequently produces scar tissue with disorganized collagen fibers, insufficient vascularization, and unresolved inflammation, yielding tissue with poor elasticity and tensile strength and increasing susceptibility to recurrence.

Current treatment strategies include conservative approaches (eccentric training, shockwave therapy, PRP injection), pharmacological interventions (e.g., corticosteroids), and surgical repair (suture, grafting, or arthroscopy) [75,76,77]. However, each strategy has significant limitations. Conservative therapies provide inconsistent results; PRP therapy lacks standardized preparation and clinical guidelines; shockwave therapy is expensive and produces highly variable outcomes; corticosteroids, though effective for short-term symptom relief, substantially increase the risk of tendon rupture; and surgical repair is often complicated by infection, adhesions, graft rejection, and suboptimal mechanical restoration.

Future research should emphasize multidimensional, personalized, and regeneration-focused therapies. For example, modulation of signaling pathways such as TGF-β3 may enhance tenocyte proliferation and promote ordered collagen deposition. Simultaneously, protein-based hydrogels and 3D biomimetic scaffolds represent promising biomaterials capable of providing mechanically compatible and bioactive microenvironments for tendon regeneration. In parallel, musculoskeletal ultrasound offers dynamic monitoring of injury progression and rehabilitation outcomes, while AI-assisted rehabilitation assessment may further improve treatment precision. By integrating sports rehabilitation, biomaterials science, and molecular therapeutics, such interdisciplinary strategies hold potential to transcend conventional structural repair, enabling functional tendon regeneration and restoring athletic capacity in both professional and recreational populations.

### 3.4. Fractures

Fractures are among the most common and destructive sports-related injuries. They may result from high-energy acute trauma, such as collisions in basketball or soccer and direct impact during falls. They may also arise from repetitive low-energy stress, as observed in long-distance running, dance, gymnastics, and military training [78]. Risk also varies by population: young athletes are more prone to acute traumatic fractures, while older individuals, due to osteoporosis, metabolic abnormalities, or reduced muscle strength, are more susceptible to fractures even during minor physical activity [79,80]. Clinically, fractures typically present with severe localized pain, swelling, deformity, and restricted mobility, and in some cases may be complicated by hemorrhage or nerve injury, leading to sensory deficits and functional loss [81]. Inadequate management may result in long-term complications such as joint stiffness, chronic pain, overuse syndromes, and persistent activity limitations. Beyond physical consequences, fractures may also have considerable psychological and social impacts. Athletes may experience anxiety, depression, loss of competitive condition, and career-threatening outcomes, whereas non-athlete patients may experience reduced independence and diminished social participation [82,83,84].

From a pathophysiological perspective, fracture healing is a complex process involving inflammation, endochondral ossification, and bone remodeling. During the acute phase, hematoma formation and inflammatory cell infiltration initiate repair, though excessive inflammation may inhibit osteogenesis. The reparative phase relies heavily on endochondral ossification and osteoblast activity, but in cases of osteoporosis or metabolic dysfunction, bone-forming capacity is significantly compromised. During remodeling, maintaining trabecular stability is crucial, yet repetitive loading may lead to microcrack accumulation, increasing the risk of refracture or malunion [43,85,86,87].

Current treatment strategies include conservative approaches (e.g., casting, traction, functional training), surgical interventions (e.g., internal fixation with plates or intramedullary nails, arthroscopic minimally invasive techniques), and regenerative medicine-based therapies (e.g., bone grafting, bone morphogenetic protein-2 [BMP-2], and stem cell transplantation) [88,89,90]. Nevertheless, these modalities face important limitations: conservative treatments are often associated with prolonged healing and high recurrence rates; surgical procedures carry risks such as infection, implant failure, and nonunion; and regenerative techniques remain constrained by limited bioactivity, poor graft integration, and potential risks of immune rejection or tumorigenesis [91,92,93].

Future directions lie in the convergence of materials science and biology. Smart biomimetic materials—such as protein hydrogel-based composite scaffolds with dynamically tunable stiffness—could provide dual functions of early mechanical support and late-stage degradation/absorption. Nanoscale drug delivery systems offer precise and controlled release of growth factors (e.g., VEGF, BMP-2) and pharmaceuticals, thereby enhancing bone regeneration efficiency. Meanwhile, musculoskeletal ultrasound provides dynamic imaging for real-time monitoring of fracture healing, enabling individualized evaluation and adaptive rehabilitation. Ultimately, multidisciplinary integration of sports rehabilitation, tissue engineering, and imaging-based monitoring may shift fracture management from simple anatomical fixation toward functional bone regeneration and comprehensive recovery.

## 4. The Role of Musculoskeletal Ultrasound in the Evaluation of Sports Injuries

Sports injuries are common in both competitive athletics and routine exercise. They may involve multiple tissues, including skeletal muscle, bone, cartilage, and tendons, and are characterized by complex and heterogeneous pathological processes [94,95]. Accurate diagnosis is the foundation for developing individualized rehabilitation strategies and guiding therapeutic interventions. Traditional imaging modalities such as X-ray, CT, and MRI provide valuable structural information; however, they remain limited in dynamic functional assessment, real-time soft tissue monitoring, and rapid bedside application [96,97,98].

In recent years, MSKUS has emerged as a pivotal diagnostic tool owing to its noninvasiveness, repeatability, high resolution, and ability to perform dynamic imaging and interventional guidance [99,100]. MSKUS can detect acute structural injuries and associated complications while also tracking functional alterations during chronic degenerative processes. In this way, it provides a practical bridge between diagnosis and therapy. A systematic summary of MSKUS applications across different tissue types is therefore important for advancing precise diagnosis and optimizing clinical intervention pathways [101,102].

### 4.1. Cartilage Injuries

In sports-related cartilage injuries, acute impact or chronic overload may cause surface fissures, microfibrillation, cartilage thinning, subchondral bone edema, and inflammatory responses [103,104]. These alterations not only compromise the buffering and lubrication functions of the joint but also directly lead to stiffness, pain, and restricted mobility. Musculoskeletal ultrasound (MSKUS), through high-frequency acoustic interactions at the cartilage–fluid interface, can precisely capture such structural changes [12]. Normal hyaline cartilage appears as a uniform hypoechoic band, whereas injury is characterized by surface irregularities, focal echogenic abnormalities, and heterogeneous thickness [105,106]. Early degenerative changes, including microfibrillation and localized edema, can be detected with sub-0.1 mm resolution, and dynamic frictional abnormalities can be directly visualized during joint movement [107]. Compared with MRI, ultrasound is more sensitive in detecting superficial cartilage lesions and early morphological alterations, while being more convenient, cost-effective, and suitable for repeated follow-up. Unlike X-ray, ultrasound provides direct visualization of soft tissues and hemodynamics without radiation exposure. Moreover, MSKUS offers unique advantages in dynamic and interventional settings: it enables real-time monitoring of cartilage–joint surface interactions during flexion, extension, and weight-bearing, and provides precise guidance and therapeutic monitoring during intra-articular injections of hyaluronic acid, platelet-rich plasma (PRP), or stem cells. Thus, MSKUS demonstrates advantages in diagnosing cartilage pathology and provides important value for dynamic monitoring and image-guided interventions [98].

**Figure 1:**
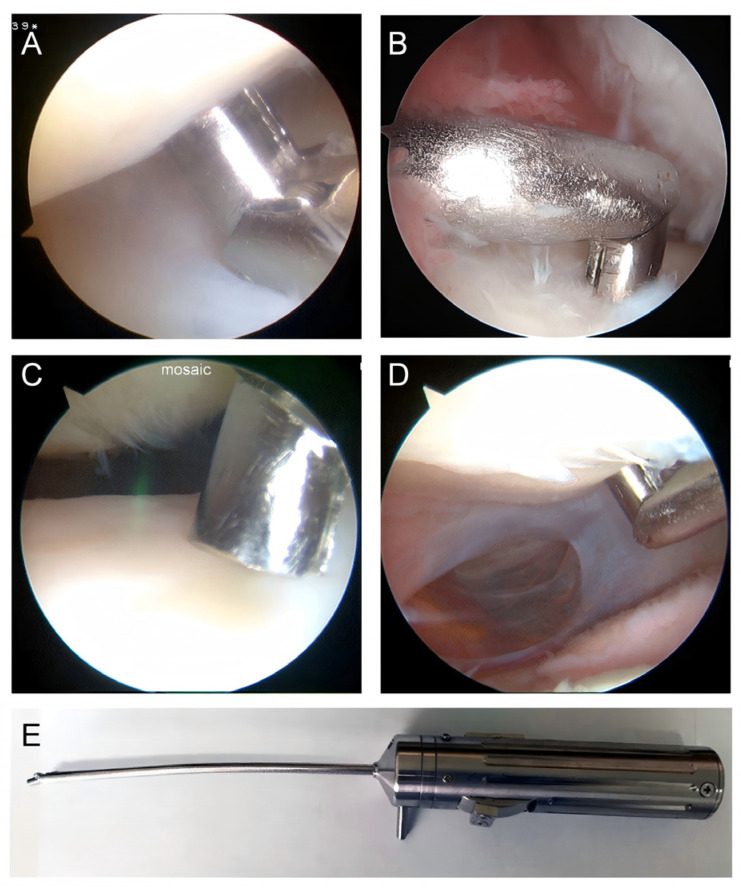
(**A**): The mechanical properties in the femoral condyle were measured; (**B**): The mechanical properties in the tibial plateau were measured; (**C**): The mechanical properties in the trochlea were measured; (**D**): The mechanical properties in the patella were measured; (**E**): The entire whole of the probe [109].

Experimental evidence further supports the role of high-frequency ultrasound elastography as a noninvasive tool for assessing intra-tissue strain and cartilage quality. Pastrama and colleagues conducted studies using bovine patellar osteochondral explants to evaluate the biomechanical response of cartilage under controlled loading. Osteochondral plugs (~1.5 × 1.5 cm^2^) were divided into three groups and subjected to compressive loads of 10 N, 40 N, and 70 N using a 4 mm hemispherical indenter (*n* = 3 per group). After 15 min of loading, samples were unloaded and imaged for 1 h using high-frequency ultrasound (18 MHz probe, Verasonics system). Axial displacement fields were computed with a speckle-tracking algorithm, and Lagrangian strain along the indentation axis was accumulated to characterize intra-tissue strain distribution during recovery. Results revealed heterogeneous strain fields across all samples: the superficial zone beneath the indenter exhibited the greatest strain, which decreased with depth; higher loads induced larger strains (maximum values at unloading: −0.51, −0.73, and −0.97 for the 10 N, 40 N, and 70 N groups, respectively) and significantly slower recovery rates (69% recovery within 10 min at 10 N, 49% at 40 N, and 43% at 70 N). Under low load, deformation was confined to the superficial layer, whereas higher loads produced more uniform strain distributions, with high-strain regions extending into deeper layers [108]. These findings highlight the accuracy of ultrasound for evaluating cartilage biomechanics. Clinical applications of ultrasound in human cartilage assessment have also demonstrated substantial promise. For example, Nakagawa and colleagues developed an ultrasound-based evaluation system capable of quantifying the hardness, surface roughness, and thickness of articular cartilage in vivo (Figure 1). This approach enables early detection of degenerative changes, discrimination among International Cartilage Repair Society (ICRS) grades (0–3), and evaluation of age and sex-related variations in normal cartilage. Experimental data showed that ICRS grade 0 cartilage exhibited significantly greater hardness than grades 1–3 (*p* < 0.0001), lower surface roughness compared with grade 1 (*p* = 0.0024), and greater thickness than grades 1–3 (*p* < 0.0001). Significant intergroup differences in hardness and thickness were observed across all grades, underscoring the system’s diagnostic sensitivity. Furthermore, its dynamic functional assessment capabilities allow real-time monitoring of stress-induced injuries [109].

However, the role of MSKUS in cartilage assessment should be interpreted in a scenario-dependent manner. Ultrasound is useful for evaluating superficial cartilage surfaces, joint effusion, synovial reaction, and dynamic cartilage–joint interactions [110], but it remains limited in assessing deep cartilage layers, subchondral bone marrow edema, and complex osteochondral lesions. In these cases, MRI still provides more comprehensive information on intra-articular structures and subchondral changes [111]. Therefore, MSKUS should be considered a complementary tool for dynamic and repeated assessment rather than a complete replacement for MRI in cartilage injury evaluation.

### 4.2. Muscle Injuries

In sports-related muscle injuries, the predominant pathological processes include acute tears, hematoma formation, chronic fibrosis, and fatty degeneration. These alterations often have a direct impact on athletic performance and rehabilitation outcomes [112]. Using high-frequency sound waves (7–15 MHz), MSKUS can clearly delineate the characteristic pennate architecture of muscle fibers [113]. In the chronic stage, ultrasound can readily identify hyperechoic fibrotic bands as well as “snowflake-like” signals caused by calcification or fatty infiltration [114]. Compared with MRI, ultrasound not only achieves resolutions up to 0.1 mm—sufficient to detect microtears smaller than 5 mm—but also provides real-time dynamic imaging. This capability enables visualization of abnormal movement patterns and functional manifestations of injury during running, jumping, or resistance activities. Therefore, for the diagnosis of sports-induced muscle injuries, MSKUS offers advantages in sensitivity and specificity and provides important value for dynamic monitoring [115].

**Figure 2:**
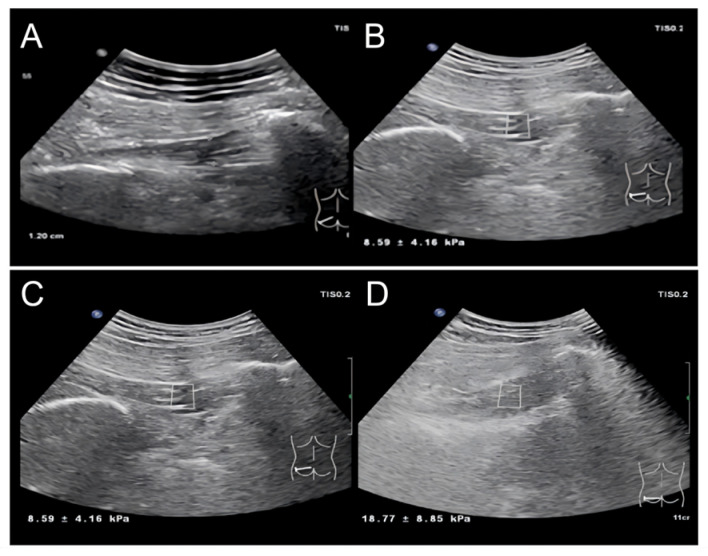
(**A**): The measured value of piriformis muscle (PM) thickness on the left side of the control group was 12.0 mm; (**B**): The Young’s modulus value of the left PM of the control group was 8.59 ± 4.16 kPa; (**C**): In control group, a female, 55 years old, the Young’s modulus value of the left piriformis muscle was 8.59 ± 4.16 kPa; (**D**): In observation group patient, a female, 53 years old, the Young’s modulus of the left piriformis muscle was 18.77 ± 8.85 kPa [116].

Xu and colleagues conducted a case–control study employing two-dimensional ultrasound combined with shear-wave elastography (SWE) to assess piriformis muscle (PM) injury in 40 patients with piriformis syndrome (PMS) and 40 healthy controls. Their findings revealed that PMS patients exhibited significantly greater PM thickness (18.23 ± 3.84 mm) and Young’s modulus (19.36 ± 9.68 kPa) compared with healthy individuals (13.70 ± 2.39 mm and 8.85 ± 2.31 kPa, respectively; *p* < 0.01), with a moderate positive correlation between the two parameters (r = 0.454, *p* < 0.05) (Figure 2). Among unilateral cases, 96.8% of affected PMs were thicker and 90.6% exhibited higher modulus values (*p* < 0.01). Combined diagnostic analysis yielded a sensitivity of 95.8%, specificity of 78.8%, and an AUC of 0.939, confirming the high diagnostic accuracy of two-dimensional ultrasound with SWE for PMS [116]. Clinically, compared with MRI, ultrasound offers greater efficiency in hematoma staging, fascial integrity assessment, and bedside interventions, while also being cost-effective and radiation-free. Danziato-Neto explored the relationship between quadriceps muscle thickness (QMT) measured by ultrasound and nutritional status in patients with high-complexity trauma admitted to the ICU. In a cohort of 30 critically injured patients, QMT was significantly lower in the moderate malnutrition group compared with other nutritional categories, and intergroup differences remained significant after excluding overweight individuals (Kruskal–Wallis: H = 7.532, *p* = 0.023). QMT correlated positively with mid-upper arm circumference (MUAC) and its adequacy index (MUACA), with Spearman coefficients of 0.557 and 0.531, respectively (*p* < 0.05). These findings suggest that ultrasound represents a valuable tool for monitoring muscle integrity in critically ill patients and can guide adjustments in nutritional therapy [117].

Moreover, as a frontline modality for superficial injuries, ultrasound has gained renewed momentum with recent advances in device technology, further enhancing its capacity for dynamic monitoring. For instance, Chen developed a wearable ultrasound imaging system integrating a customized T-shaped transducer, dual-directional shear-wave elastography (DDSWE), and an optical tracking system for real-time assessment of shoulder muscle mechanics, primarily the deltoid. This device enables simultaneous acquisition of longitudinal (along fibers) and transverse (across fibers) shear-wave velocities (SWVs) without the need to rotate the transducer, while synchronously recording 3D shoulder positions. Experimental validation demonstrated measurement bias of ~6% and precision of ~1.2% for SWVs, with optical tracking yielding coefficients of determination (R^2^) of 0.99–1.0. In vivo testing showed that as shoulder abduction increased from 0° to 60°, transverse SWV rose from 2.24 to 3.35 m/s, while longitudinal SWV increased from 2.95 to 5.95 m/s, with a ratio of 1.3–1.8 and correlation coefficients of 0.83 (longitudinal) and 0.77 (transverse). These results confirm the system’s ability to quantitatively assess muscle dynamics and anisotropy, thereby highlighting the advantages of ultrasound in dynamic functional evaluation [118].

Despite these advantages, MSKUS evaluation of muscle injuries is influenced by several technical and biological factors. Image interpretation may vary with probe pressure, scanning angle, muscle contraction state, hematoma stage, lesion depth, and examiner experience. Although ultrasound is highly useful for superficial muscle tears, hematoma monitoring, and dynamic assessment during contraction [119], MRI may still be required for deep muscle injuries, diffuse edema, complex pelvic or hip-region lesions, or uncertain differential diagnosis [120]. Therefore, MSKUS is most valuable when used as a rapid, repeatable, and function-oriented modality in combination with clinical examination and complementary imaging when necessary.

### 4.3. Tendon Injuries

In sports-related tendon injuries, the pathological changes typically include acute rupture, partial fiber disruption, chronic degeneration, and disorganized collagen alignment [121]. These alterations compromise joint stability, reduce the efficiency of force transmission, and limit athletic performance. By leveraging the acoustic reflection characteristics of dense collagen fibers, MSKUS can clearly visualize the hyperechoic parallel fibrillar pattern of healthy tendons [122]. In contrast, characteristic alterations appear in injured tendons: acute complete ruptures present with fiber discontinuity and the classic “horse-tail sign,” partial tears appear as fissure-like hypoechoic defects that may exhibit the “falling-leaf sign” during dynamic traction, while chronic degeneration is characterized by fusiform thickening, calcification, and neovascularization signals detectable with power Doppler [123]. Compared with MRI, ultrasound may be more sensitive for detecting superficial tendon abnormalities and small tears, and it enables real-time functional assessment of injury sites during joint movement. Unlike X-ray imaging, ultrasound not only depicts soft-tissue structures but also provides hemodynamic and tissue activity information without radiation exposure [124]. More importantly, musculoskeletal ultrasound combines dynamic and interventional advantages: it allows direct evaluation of tendon mechanical stability and abnormal movement during running, jumping, or resistance exercises, and it provides precise guidance during treatments such as platelet-rich plasma (PRP) injections, stem cell implantation, or percutaneous needle therapy, thereby enhancing both safety and efficacy [125,126]. Thus, in sports-related tendon disorders, ultrasound provides diagnostic advantages and has distinctive value for dynamic assessment and precise intervention.

**Figure 3:**
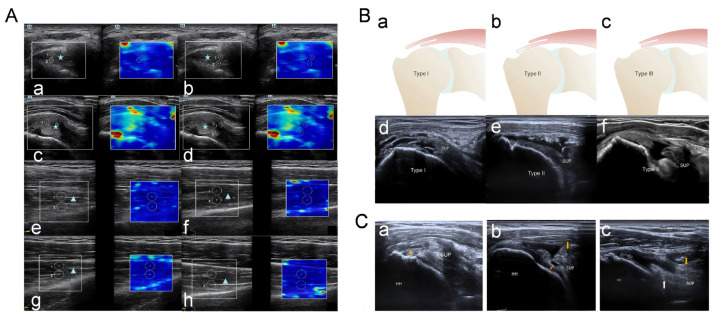
(**A**): Ultrasound image of the supraspinatus tendon and muscle measured by SWE. (**a**,**b**): Natural position SWE measured SWV at four sites of the supraspinatus tendon; (**c**,**d**): modified crass position SWE measured SWV at four sites of the supraspinatus tendon; (**e**,**f**): Natural position SWE measured SWV at four sites of the supraspinatus muscle; (**g**,**h**): modified crass position SWE measured SWV at four sites of the supraspinatus muscle. Pentagram: residual tendon after tear of supraspinatus tendon. Triangle: supraspinatus muscle [127]. (**B**): Graphical representations and ultrasound images of three shapes of delaminated tears. (**a**,**d**): Type I tears, showing greater retraction of articular layer; (**b**,**e**): type II tears, showing greater retraction of bursal layer; (**c**,**f**): type III tears, showing equal retraction of both layers. (**C**): Ultrasonographic signs of delaminated tears of supraspinatus tendon in longitudinal plane. (**a**): Anechoic horizontal linear splitting of tendon (yellow triangle); (**b**): Anechoic horizontal linear splitting of tendon (yellow triangle) with the retraction of bursal layer (yellow arrow); (**c**): Bursal layer (yellow arrow) more retract than articular layers (white arrow). HH, humerus head; SUP, supraspinatus [130].

Chen and colleagues investigated the accuracy of shear-wave elastography (SWE) for preoperative evaluation of residual tendon quality in rotator cuff repair (Figure 3A). Their study included 89 surgical shoulders and 40 control shoulders, with measurements of shear-wave velocity (SWV) in the supraspinatus tendon and muscle. Ratios of tendon-to-deltoid SWV (SWV-RT) and muscle-to-deltoid SWV (SWV-RM) were calculated and compared with arthroscopic scores (gold standard). Results showed significant negative correlations between arthroscopic scores and both SWV-RT (R = −0.722 to −0.884) and SWV-RM (R = −0.569 to −0.689), with values significantly lower in surgical shoulders (*p* < 0.05). SWE also demonstrated excellent intra- and inter-observer reliability (κ = 0.848 and 0.697, respectively). These findings indicate that SWE provides valuable predictive information on residual tendon quality and can assist in preoperative assessment for rotator cuff repair [127]. Furthermore, ultrasound is also highly effective in evaluating tendon healing after injury. It enables real-time monitoring of tendon elasticity and structural changes, thereby allowing physicians to more accurately assess healing progression, tailor individualized rehabilitation strategies, and ultimately improve treatment outcomes and patient satisfaction. In another study, Zheng applied ultrasound for noninvasive monitoring of tendon healing by quantifying elasticity, mechanical properties, and microstructural changes through parameters such as average integrated backscatter (AIB), spectral centroid shift (SCS), and frequency slope of the apparent backscatter (FSAB). Results demonstrated that AIB correlated strongly with ultrasonic elastic properties (in vitro and in vivo shear modulus: r = −0.71 and −0.70) and weakly with mechanical properties (in vitro and in vivo tensile modulus: r = −0.43 and −0.50). During the healing process, AIB, SCS, and FSAB values decreased progressively (e.g., in the MC group at day 28, AIB = −69.1 ± 13.4 dB, approaching the NC group value of −68.4 ± 11.2 dB). The LT group showed greater improvement than the MC group (e.g., LT group day 28 AIB = −71.8 ± 5.3 dB vs. MC group), demonstrating the effectiveness of ultrasound in evaluating therapeutic outcomes [128].

A key advantage of ultrasound over MRI lies in its dynamic functional assessment [129]. For example, in rotator cuff tears, ultrasound can visualize lesion manifestations at specific joint positions, capture abnormal tendon gliding in real time, and achieve ultra-high-resolution imaging of superficial structures such as the extensor tendons of the hand or the Achilles tendon. Wang investigated the role of real-time dynamic ultrasound in diagnosing delaminated rotator cuff tears (Figure 3B), emphasizing its ability to capture tendon motion changes dynamically, thereby overcoming the limitations of static imaging. By correlating imaging findings with clinical signs, ultrasound can detect subtle lesions that are otherwise difficult to identify, playing a crucial role in diagnosis (Figure 3C). When validated against arthroscopy, dynamic ultrasound achieved a sensitivity of 72.0% and specificity of 96.7% for delaminated tears, with sensitivities of 56%, 72%, and 100% for Type I, II, and III tears, respectively, and specificities of 80%, 83%, and 98%. Three characteristic features—anechoic horizontal fissures, asymmetric layer retraction, and thinning of the affected tendon—demonstrated specificities of 100.0%, 100.0%, and 97.9%, respectively. These findings highlight the clinical effectiveness of ultrasound in dynamic evaluation of rotator cuff pathology [130].

Nevertheless, MSKUS-based tendon evaluation also has important limitations. Tendon anisotropy, probe orientation, joint position, and examiner experience can substantially influence image quality and diagnostic interpretation. Although ultrasound is highly effective for superficial tendons, dynamic tendon gliding, Doppler vascularity, and image-guided interventions [131], MRI remains valuable for complex deep tendon lesions, intra-articular extension, associated bone marrow edema, or preoperative mapping. Moreover, SWE and Doppler parameters still lack universally accepted thresholds for defining healing quality [132]. Therefore, ultrasound findings should be interpreted together with symptoms, functional testing, and, when necessary, MRI or surgical findings.

### 4.4. Fracture Injuries

In sports-related fractures, common pathological changes include cortical discontinuity caused by acute high-energy impact, trabecular microcracks resulting from stress injury, and subperiosteal hematoma accompanied by adjacent soft tissue damage. These changes may directly compromise joint stability and functional recovery [133,134]. MSKUS, leveraging the acoustic properties of high-frequency sound waves at the bone–soft tissue interface, can accurately depict the characteristic features of fractures: normal cortical bone appears as a continuous hyperechoic line with acoustic shadowing, whereas fractures manifest as cortical interruptions with a “step-off sign,” multilayer fractures indicated by the “double cortex sign,” and hypoechoic subperiosteal hematoma bands (>2 mm) [135]. Moreover, MSKUS can also identify associated soft tissue injuries such as tendon displacement or hematoma infiltration. Compared with radiography, ultrasound demonstrates superior sensitivity in detecting occult fractures, stress fractures, and pediatric fractures, while avoiding ionizing radiation; compared with MRI, it offers flexible operation and bedside availability, making it particularly suitable for emergency and on-field rapid screening [48]. More importantly, MSKUS provides dynamic and interventional value: it enables real-time assessment of fracture stability and healing progression, and offers precise guidance during closed reduction, fracture-site injections, or rehabilitation interventions. Thus, MSKUS may provide high sensitivity for detecting sports-related fractures and has important value in real-time monitoring and interventional management [136].

**Figure 4:**
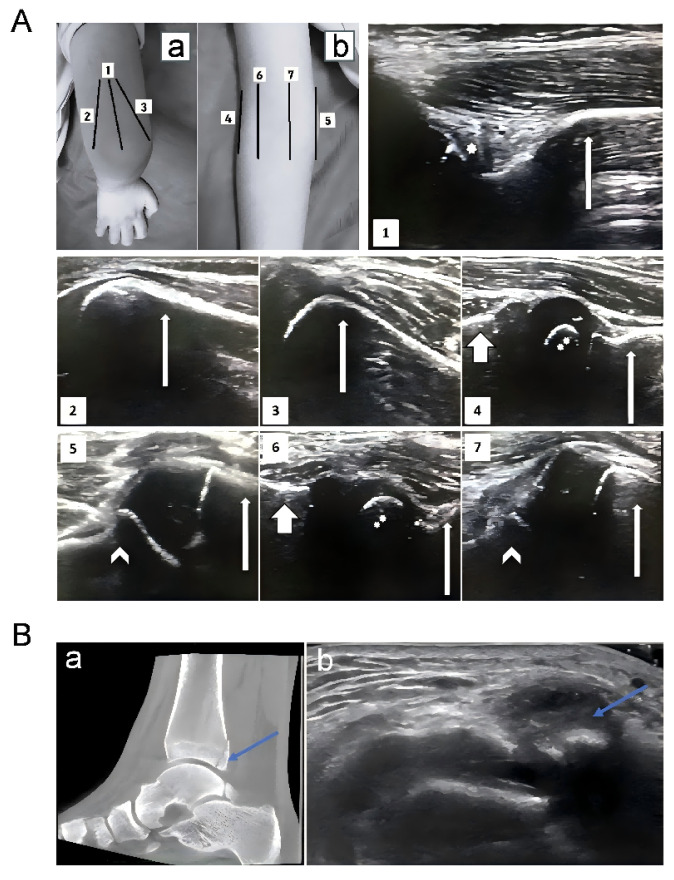
(**A**): (**a**): Elbow flexion position and posterior of humerus; (**b**): Elbow extension position anterior of elbow, (1): Dorsomedian aspect, (2): Dorsoulnar aspect, (3): Dorsoradial aspect, (4): Lateral aspect, (5): Medial aspect, (6): Ventroradial aspect, (7): Ventroulnar aspect, asterisk: posterior fat pad, double asterisks: Capitellum, long arrow: Humerus, Short arrow: Radius, arrowhead: Ulna [137]. (**B**): A fracture of the posterior malleolus of the tibia seen on CT (**a**) and ultrasound with a deltoid ligament rupture (**b**) in the same patient (arrow) [138].

In the study by Korgan, a comparative analysis was conducted to evaluate the diagnostic accuracy of ultrasound in pediatric elbow injuries (Figure 4A). Among 128 patients, 122 completed the study, and ultrasound achieved a definitive fracture diagnosis in 94.9% of cases. Compared with discharge diagnosis, its sensitivity was 94.9% (95% CI: 82.7–99.37%), specificity 83.13% (95% CI: 73.32–90.46%), accuracy 0.86, positive likelihood ratio 5.62, negative likelihood ratio 0.06, positive predictive value 72.55% (95% CI: 61.98–81.08%), and negative predictive value 97.18% (95% CI: 89.91–99.26%). Regarding reduction in unnecessary radiographs, among 56 low-risk patients with no motion limitation and normal ultrasound findings, only one was ultimately diagnosed with fracture, reducing unnecessary radiographs by 46% [137]. Furthermore, compared with X-ray, the core advantage of MSKUS lies in its high sensitivity for occult fractures (e.g., incomplete rib fractures, subtle scaphoid cracks), its ability to simultaneously visualize soft tissue involvement (e.g., nerve entrapment), and its radiation-free nature, making it especially applicable in children, pregnant women, and superficial bones (e.g., distal radius, tibial crest). In Allen’s study, MSKUS was confirmed as an important complementary tool in diagnosing occult fractures, particularly in acute ankle trauma with negative radiographs (Figure 4B). Among 100 patients with normal X-rays, ultrasound detected 7 major fractures (all CT-confirmed, no false positives) and 40 minor avulsion fractures. Although complementary with CT in detecting minor avulsions (4 identified by ultrasound only, 6 by CT only), ultrasound additionally revealed 83 anterior talofibular ligament and 39 deltoid complex injuries, and could be integrated with cone-beam CT to improve diagnostic accuracy. All 7 patients with major fractures required referral for further treatment [138]. Similarly, Snelling demonstrated the superiority of ultrasound over radiography in detecting pediatric distal forearm fractures. In terms of diagnostic accuracy, the ultrasound group achieved higher overall accuracy (97.8%) compared to the radiography group (83.0%). For both “buckle” and “other” fracture subtypes, diagnostic accuracy was consistently higher in the ultrasound group. In terms of specificity, ultrasound outperformed radiography in diagnosing both “any” and “other” fractures, with differences of 18.4% (95% CI: 5.8–31.0%) and 8.3% (95% CI: 1.7–15.0%), respectively. Clinically, only one participant in the ultrasound group underwent unnecessary casting, compared to 10 in the radiography group, with an average immobilization duration of 28 days. These findings highlight that, beyond the advantage of radiation-free imaging, MSKUS provides superior diagnostic accuracy, specificity, and clinical guidance in the detection of superficial pediatric fractures, offering a more reliable basis for diagnosis of distal forearm injuries [139].

Although MSKUS can improve the detection of superficial cortical disruption, subperiosteal hematoma, and associated soft-tissue injury, it is not suitable for all fracture scenarios [140]. Its diagnostic value decreases for deep bones, complex intra-articular fractures, subtle trabecular injury, bone marrow edema, and regions obscured by overlying bone or gas. Radiography, CT, or MRI remains necessary for fracture classification, surgical planning, and evaluation of deep or complex lesions [141]. Therefore, MSKUS should be positioned as a radiation-free complementary tool for selected superficial or occult fractures, bedside screening, and follow-up monitoring rather than as a universal substitute for conventional imaging.

### 4.5. MSKUS-Guided Hydrogel Therapy: Delivery, Localization, and Monitoring

The MSKUS–hydrogel strategy can be better understood as a continuous process linking lesion evaluation, hydrogel selection, targeted delivery, post-intervention monitoring, and rehabilitation feedback. In this process, MSKUS is not limited to identifying the injured tissue; it may also help define the lesion phenotype [142], guide hydrogel formulation and delivery route, confirm material localization, and provide follow-up information on tissue response and functional recovery. Therefore, the integration between MSKUS and protein-based hydrogels can be discussed through four interconnected stages: pre-intervention assessment, hydrogel selection and delivery planning [143], ultrasound-guided administration, and longitudinal monitoring with rehabilitation feedback.

Beyond its diagnostic value, MSKUS may also serve as a practical link between injury assessment and localized therapeutic intervention. In sports injury management, accurate recognition of lesion location, tissue depth, structural disruption, vascular response, and mechanical properties is essential not only for diagnosis, but also for determining the most appropriate therapeutic strategy [119]. Since protein-based hydrogels can be designed as injectable matrices, adhesive patches, drug-loaded systems, or tissue-specific scaffolds, MSKUS-derived information may help clarify whether the injured site requires superficial coverage, image-guided injection, intra-articular delivery, or defect-adjacent placement [144,145].

More specifically, baseline MSKUS can be used to define the lesion phenotype before hydrogel intervention, including injury type, lesion depth, defect size, structural continuity, hematoma or effusion, Doppler vascularity, and tissue stiffness. These imaging findings can then be translated into hydrogel-related decisions. For example, a superficial muscle tear with a visible defect may be more suitable for an injectable defect-filling hydrogel [146], whereas a tendon lesion with peritendinous inflammation may require an anti-inflammatory or anti-adhesive hydrogel [142]. Joint effusion, synovial reaction, and superficial cartilage irregularity may support intra-articular or defect-adjacent hydrogel delivery, whereas deep osteochondral lesions may require complementary MRI or arthroscopic evaluation before hydrogel intervention.

For muscle and tendon injuries, MSKUS can identify fiber discontinuity, hematoma formation, peritendinous inflammation, scar development, and changes in tissue stiffness. These findings may provide useful information for selecting hydrogels with anti-inflammatory, pro-regenerative, collagen-remodeling, or mechanically supportive functions [147,148]. For cartilage or joint-related injuries, ultrasound assessment of synovial reaction, joint effusion, cartilage surface irregularity, and periarticular soft-tissue changes may help determine whether hydrogel systems with lubricating, chondrogenic, anti-inflammatory, or sustained-release properties are more suitable [149,150]. In this way, imaging assessment may help translate local pathological features into more targeted hydrogel-based repair strategies. This step is important because different sports injuries require different material functions. Muscle injuries require defect filling, elasticity, and support for myofiber regeneration; tendon and ligament injuries require anti-adhesion, collagen remodeling, and mechanical support; cartilage and joint-related injuries require lubrication, anti-inflammatory regulation, and chondrogenic stimulation [151]; and bone or tendon–bone interface injuries may require osteogenic support, interfacial integration, and compatibility with mechanical stabilization [152]. Therefore, MSKUS-derived lesion information should be linked directly with hydrogel formulation, delivery route, and follow-up endpoints rather than being used only for descriptive diagnosis.

During intervention, ultrasound guidance may further improve the precision of hydrogel delivery and localization. For injectable hydrogels, real-time ultrasound can help determine the needle path, avoid adjacent vessels and nerves, and guide the material toward the intended lesion area, such as a muscle tear, peritendinous region, joint cavity, or defect-adjacent space [153]. Immediate post-injection imaging may also help confirm whether the hydrogel has been distributed within or around the target site. For topical or adhesive hydrogel systems, MSKUS may assist in evaluating lesion depth and tissue involvement, thereby helping to judge whether surface application is likely to reach the therapeutic target or whether a deeper delivery strategy may be required. In a practical procedure, MSKUS can first identify the target tissue and surrounding anatomical structures, then guide the needle tip or applicator toward the lesion under real-time visualization [154]. During administration, ultrasound can help determine whether the hydrogel fills the intended defect, spreads along the desired tissue plane, or diffuses into non-target spaces. This step is particularly important for injectable hydrogels because inaccurate placement may lead to insufficient defect filling, uneven therapeutic distribution, leakage, or irritation of adjacent tissues [142].

The delivery strategy should also be adapted to the injury type. In muscle injuries, MSKUS may help identify the tear gap, hematoma boundary, and viable muscle margins, thereby guiding the hydrogel into the defect or peri-lesional region. In tendon and ligament injuries, ultrasound guidance may support peritendinous, intratendinous, or interface-adjacent delivery while reducing the risk of excessive intratendinous pressure or injury to surrounding neurovascular structures. In cartilage and joint-related injuries, ultrasound may assist intra-articular injection and help confirm joint effusion, synovial reaction, and periarticular soft-tissue status, although deep cartilage or osteochondral defects may still require MRI or arthroscopy for complete evaluation [155]. In superficial bone or tendon–bone interface injuries, ultrasound may help monitor cortical surface changes, periosteal reaction, and adjacent soft-tissue repair, whereas CT or MRI may remain necessary for deep bone healing assessment [140].

After hydrogel-based intervention, MSKUS may contribute to follow-up evaluation by monitoring both local tissue response and, in some cases, material behavior. Depending on hydrogel echogenicity, implantation depth, and the contrast between the hydrogel and surrounding tissues, ultrasound may directly or indirectly reflect hydrogel retention, displacement, volume change, or degradation-related changes [156]. More importantly, repeated MSKUS assessment can be used to observe changes in swelling, vascular signals, tissue continuity, scar formation, stiffness recovery, joint effusion, and functional movement. These parameters may provide useful feedback on whether the local repair process is progressing appropriately. For post-intervention monitoring, it is useful to distinguish between material-related monitoring and tissue-response monitoring [154]. If the hydrogel has sufficient acoustic contrast, MSKUS may allow repeated observation of material localization, boundary clarity, volume change, displacement, fragmentation, or degradation-related morphological changes. However, direct visualization of hydrogel degradation should not be assumed for all protein-based hydrogels. Many hydrogels may become difficult to distinguish from adjacent soft tissues after swelling, tissue infiltration, or partial degradation [157]. In such cases, MSKUS should be used primarily as an indirect monitoring tool.

Indirect therapeutic-response monitoring may include evaluation of local edema, Doppler vascularity, tissue continuity, scar formation, stiffness recovery, joint effusion, and dynamic functional movement. For example, reduced edema and Doppler signal may suggest attenuation of local inflammation; improved fiber continuity or tendon gliding may indicate structural repair; progressive normalization of SWE-derived stiffness may reflect matrix remodeling [132]; and decreased joint effusion during follow-up may suggest improved joint homeostasis. These imaging indicators should be interpreted together with pain scores, functional tests, rehabilitation tolerance, and, when necessary, MRI, CT, arthroscopy, histological analysis, or biomechanical testing. In this way, MSKUS can become part of a feedback process in which imaging findings are used not only to document repair, but also to guide rehabilitation intensity and return-to-sport decision-making [158].

Several clinically relevant studies have begun to illustrate how ultrasound guidance can support localized biomaterial delivery and treatment monitoring in musculoskeletal conditions, providing useful references for the future development of MSKUS-guided hydrogel-based strategies in sports injury repair. In a randomized controlled trial involving 168 patients with hand tendon injuries, Yin et al. compared postoperative ultrasound-guided carboxymethyl chitosan hydrogel injection with intraoperative hydrogel application. At 12 months, the ultrasound-guided postoperative injection group showed greater total active motion than the intraoperative application group (209° vs. 189°; mean difference, 20°; *p* = 0.006), accompanied by better early pain relief and hand function recovery. These findings suggest that ultrasound-guided hydrogel delivery and optimized intervention timing may improve functional outcomes in tendon-related conditions. This evidence supports the view that image-guided hydrogel delivery may influence not only localization accuracy, but also functional recovery when the timing and target of intervention are optimized [142].

In another pilot clinical study, Latini et al. used two ultrasound-guided injections of low-molecular-weight peptides derived from hydrolyzed bovine collagen in 21 patients with ultrasound-detected partial supraspinatus tendon tears. At 12 weeks, VAS pain scores decreased from 63 ± 20.5 to 37 ± 23.3, and SPADI total scores improved from 80.6 ± 21.5 to 50.3 ± 23.5. Improvement in VAS pain was observed in 81% of patients, while improvement in SPADI scores was observed in 85.7% of patients. The injections were generally well tolerated, with only one patient showing progression of tendon damage during follow-up [159]. Together, these studies suggest that image-guided localization, targeted biomaterial delivery, and follow-up evaluation are clinically relevant components for future MSKUS-guided hydrogel-based strategies. Although these studies were not designed specifically to establish a universal MSKUS–hydrogel protocol for sports injuries, they provide clinically relevant support for three key components of the proposed framework: image-based lesion identification, targeted local biomaterial delivery [142], and follow-up assessment of therapeutic response. Future studies should further compare MSKUS-guided hydrogel administration with non-guided delivery and conventional treatments using standardized endpoints, such as hydrogel retention, degradation behavior [157], imaging-based tissue repair, pain scores, functional scales, return-to-sport time, and adverse events. Based on the above discussion, the proposed MSKUS-guided hydrogel therapy pathway was further organized by linking each clinical stage with imaging inputs, hydrogel-related decisions, and follow-up indicators (Table 2).

Taken together, MSKUS and protein-based hydrogels can be integrated through a continuous clinical pathway that links lesion assessment, hydrogel selection, image-guided delivery [142], post-treatment monitoring, and rehabilitation adjustment. In this pathway, MSKUS first helps define the lesion phenotype, including structural disruption, tissue depth, vascular response, stiffness, and functional changes. These findings can then guide the selection and delivery of appropriate hydrogel systems [142,157]. After intervention, follow-up imaging can be used to monitor hydrogel-related changes and local tissue responses, while repeated structural and functional feedback may support rehabilitation progression and return-to-sport decision-making. This integrated logic provides a more practical basis for combining dynamic imaging evaluation with localized biomaterial-assisted repair in sports injury management [157].

### 4.6. Limitations and Critical Considerations of MSKUS in Imaging-Guided Hydrogel Therapy

Although MSKUS provides a useful link between injury assessment and localized hydrogel-based intervention, its application in imaging-guided hydrogel therapy should be interpreted cautiously [181]. The value of MSKUS lies mainly in dynamic assessment, lesion localization, image-guided delivery, and repeated follow-up in selected sports injury scenarios. However, it should not be regarded as a universal replacement for MRI, CT, or radiography, nor should it be considered sufficient by itself to validate hydrogel therapeutic efficacy [142].

First, MSKUS remains highly operator-dependent. Image quality, lesion identification, needle visualization, and interpretation of post-intervention changes can be influenced by probe positioning, probe pressure, scanning angle, anisotropy, patient posture, transducer frequency, and examiner experience [148,149]. In MSKUS-guided hydrogel therapy, these factors may have direct therapeutic consequences. Inaccurate lesion localization or needle-path planning may lead to inappropriate hydrogel placement, uneven material distribution, leakage into non-target spaces, or inconsistent interpretation of repair outcomes. Therefore, standardized scanning protocols, structured operator training, and interobserver reliability assessment are necessary before MSKUS-guided hydrogel strategies can be reliably compared across studies or clinical centers [182].

Second, the anatomical and physical limitations of ultrasound may restrict its role in hydrogel delivery and monitoring. MSKUS is generally more suitable for superficial muscles, tendons, ligaments, and periarticular soft tissues, whereas deep intra-articular structures, bone marrow edema, complex osteochondral lesions, and tissues obscured by bone or gas remain difficult to evaluate comprehensively [183]. In these scenarios, MRI, CT, or radiography may still be required for structural confirmation, treatment planning, or differential diagnosis [158]. Similarly, for hydrogel monitoring, ultrasound visibility depends on hydrogel echogenicity, implantation depth, surrounding tissue contrast, and material degradation behavior [184]. Some protein-based hydrogels may not be clearly distinguishable from adjacent soft tissues after swelling, tissue infiltration, or partial degradation. In such cases, ultrasound may provide only indirect information through changes in swelling, vascular signals, tissue continuity, stiffness, effusion, or dynamic movement rather than direct visualization of hydrogel degradation [157].

Third, although Doppler ultrasound and shear wave elastography may provide useful information on vascular response and tissue stiffness, their quantitative parameters remain sensitive to device settings, acquisition protocols, probe pressure, joint position, muscle contraction state, and patient-related factors [185]. The lack of universally accepted diagnostic thresholds limits their use as standardized outcome measures for evaluating hydrogel-assisted repair [186]. This limitation is particularly relevant for longitudinal rehabilitation monitoring, where small changes in tissue stiffness or vascularity may be interpreted differently if imaging conditions are not consistent [132].

Therefore, future MSKUS-guided hydrogel studies should not rely only on successful material localization or short-term symptom improvement. More comprehensive evaluation should include quantitative imaging reliability, hydrogel retention or degradation behavior, adverse events, tissue-level repair quality, rehabilitation-related mechanical performance, patient-reported outcomes, and return-to-sport criteria. In addition, MSKUS should be combined with complementary imaging, biomechanical testing, and functional rehabilitation outcomes when necessary [154,157]. Establishing standardized scanning protocols, predefined imaging endpoints, operator training systems, and material-specific monitoring strategies will be essential for translating MSKUS-guided hydrogel therapy from conceptual feasibility to reproducible clinical application.

## 5. Applications of Hydrogels in the Treatment of Sports Injuries

Following lesion identification and functional assessment, the next key issue in sports injury management is how to provide tissue-specific repair support. The repair of sports-related injuries is strongly influenced by the biological characteristics and mechanical environment of the damaged tissue [187]. Muscle, tendon, cartilage, ligament, and bone differ markedly in cellular composition, extracellular matrix organization, vascular supply, loading pattern, and intrinsic regenerative capacity [188]. Therefore, the application of protein-based hydrogels in sports injury repair should not be understood only from the perspective of general biocompatibility or drug-loading ability. Instead, their therapeutic performance depends on whether their molecular composition, network structure, degradation behavior, and mechanical properties can match the specific repair requirements of the injured tissue [189]. In this context, the key question is not simply whether a hydrogel can support cell survival or carry bioactive molecules, but whether its structural and physicochemical properties can be translated into tissue-specific biological responses and functional repair [190].

From a structure–property perspective, protein-based hydrogels provide different biological effects according to the protein source and network architecture [191]. Collagen-based hydrogels can partially mimic the fibrillar extracellular matrix and provide cell-recognition sites that support cell adhesion, migration, and tissue integration. However, their relatively weak mechanical stability often requires further crosslinking or reinforcement when they are used in load-bearing tissues [14,192,193]. Gelatin and gelatin methacryloyl (GelMA) retain bioactive motifs derived from collagen and offer good processability, tunable photocrosslinking, and compatibility with cell encapsulation or three-dimensional bioprinting. These features make them useful for constructing cell-supportive microenvironments, although their mechanical strength and long-term stability may still be insufficient for tendon, cartilage, or osteochondral repair without additional modification [194]. Silk fibroin hydrogels, in contrast, are characterized by β-sheet-rich structures that contribute to higher mechanical strength, slower degradation, and improved structural stability, making them attractive for mechanically demanding repair environments [195]. Nevertheless, excessive structural stabilization may delay degradation and restrict cell infiltration, indicating that mechanical reinforcement must be balanced with biological remodeling. Thus, different protein sources do not only provide different material “types”; they also determine distinct trade-offs among cell affinity, mechanical strength, degradation rate, permeability, and tissue integration [151,196].

More broadly, the therapeutic performance of protein-based hydrogels is governed by a structure–property–function relationship [143]. Protein source and molecular motifs first determine the basic biological interactions between the material and cells. Crosslinking density, pore architecture, swelling behavior, stiffness, adhesion, and degradation rate then determine how the hydrogel behaves within injured tissues. These material properties further regulate cell adhesion, migration, mechanotransduction, inflammatory regulation, extracellular matrix deposition, and tissue remodeling [143,190,196]. For example, a highly crosslinked network may improve stiffness and degradation resistance, but it may also reduce pore size, nutrient diffusion, cell infiltration, and bioactive-factor release. In contrast, a loosely crosslinked network may support cellular infiltration and molecular diffusion, but it may degrade too rapidly or fail to provide sufficient mechanical support under repeated loading. Therefore, hydrogel design for sports injury repair requires a balance among mechanical support, biological remodeling, and spatiotemporal release behavior [143,151,190].

In addition to structural support, protein-based hydrogels can act as local delivery platforms for cells, growth factors, anti-inflammatory agents, extracellular vesicles, or small-molecule therapeutics [197]. The release behavior of these bioactive components is controlled by multiple mechanisms, including diffusion through the hydrated network, swelling-regulated transport, mesh-size restriction, electrostatic interaction, affinity binding, and degradation-mediated release [198]. In injury microenvironments, inflammatory enzymes, oxidative stress, pH changes, and repeated mechanical loading may further influence hydrogel degradation and therapeutic release [199]. Therefore, an ideal hydrogel system should not only prolong local retention, but also coordinate release kinetics with the temporal sequence of tissue repair, including early inflammation regulation, cell recruitment, matrix deposition, and late-stage remodeling. If release occurs too rapidly, anti-inflammatory agents, growth factors, or extracellular vesicles may be lost before the repair cascade is established; if release is too slow, the hydrogel may fail to match the timing of inflammation resolution, cell recruitment, and matrix maturation [200].

Mechanical compatibility is another critical factor for sports injury repair. If the hydrogel is substantially softer than the native tissue, it may fail to provide sufficient load transfer, defect filling, or structural support. Conversely, an overly stiff hydrogel may cause stress shielding, interface delamination, impaired mechanotransduction, or local tissue irritation [151,190,201]. This issue is particularly important in tendon, cartilage, tendon–bone interface, and bone-related injuries, where mechanical loading directly regulates tissue organization and functional recovery [152]. Long-term stability should also be considered because repeated joint motion, swelling, enzymatic degradation, and rehabilitation loading may induce fatigue damage or premature loss of hydrogel function [202]. Therefore, the mechanical design of hydrogels should be evaluated not only by initial modulus or compressive strength, but also by fatigue resistance, viscoelastic behavior, degradation–repair matching, and stability under rehabilitation-related loading [203].

Potential immunogenicity and biosafety concerns further limit the direct translation of protein-based hydrogels. Natural protein sources may show batch-to-batch variability, and incomplete purification may introduce immunogenic residues or biologically active contaminants [204]. Chemical modification and crosslinking can improve hydrogel stability, but residual monomers, photoinitiators, crosslinkers, or degradation byproducts may affect local tissue responses [205]. These safety concerns are also linked to structure–property relationships, because changes in protein source, molecular weight, crosslinking chemistry [196], sterilization conditions, or degradation pathway [189] may alter hydrogel mechanics, swelling, release behavior, degradation products, and cellular responses. Therefore, the following section discusses protein-based hydrogels in different sports injury scenarios not only according to their application potential, but also in relation to their structural basis, release behavior, mechanical matching, long-term stability, and biological safety (Table 3). This organization is intended to connect material structure with therapeutic mechanisms and to clarify why different injured tissues require different hydrogel design strategies [151].

### 5.1. Cartilage Injuries

Protein-based hydrogels, owing to their biomimetic nature and tunable functionality, exhibit unique advantages in the repair of sports-related cartilage injuries [230]. Unlike traditional materials that provide only mechanical support, these hydrogels can replicate the biochemical and structural microenvironment of the native cartilage extracellular matrix, thereby promoting cell adhesion, proliferation, and differentiation, which facilitates the regeneration of hyaline cartilage [19]. However, the therapeutic value of protein-based hydrogels in cartilage repair depends not only on their general biomimetic properties, but also on whether their structure and mechanics match the specific requirements of cartilage tissue [231]. For cartilage repair, the key structure–property requirements include compressive stiffness, hydration-mediated lubrication, controlled degradation, and the ability to maintain the chondrocyte phenotype [231,232]. These properties influence therapeutic performance by regulating mechanotransduction, extracellular matrix deposition, inflammatory cytokine exposure, and integration with subchondral bone [232].

At the same time, their adjustable mechanical properties allow them to maintain elasticity and resilience compatible with native cartilage under dynamic loading conditions, thereby enhancing the integration between newly formed and host tissues [233]. An appropriate mechanical balance is particularly important because cartilage is exposed to repeated compression and shear stress during sports activities [231]. A hydrogel that is too soft may fail to provide sufficient load distribution or defect stabilization, whereas an overly stiff hydrogel may impair local mechanotransduction, increase interfacial stress, or interfere with matrix remodeling. Therefore, cartilage-oriented hydrogels should not only support chondrogenic activity, but also provide a mechanically and biochemically favorable microenvironment for hyaline-like matrix formation [143].

**Figure 5:**
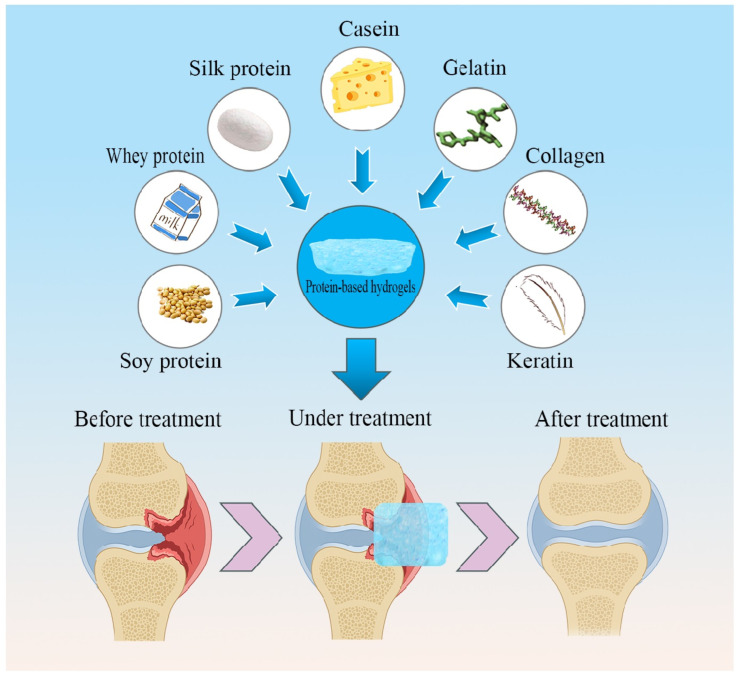
Schematic diagram of protein-based hydrogel applied to cartilage repair.

More importantly, the porous network of protein-based hydrogels provides strong capacity for drug and bioactive molecule delivery, enabling the localized and sustained release of growth factors, stem cells, or anti-inflammatory agents, thus helping to overcome the intrinsic “low regenerative capacity” of cartilage (Figure 5) [234]. The pore architecture, swelling behavior, and degradation rate of these hydrogels further determine nutrient exchange, cell infiltration, bioactive-factor diffusion, and the timing of matrix replacement [149]. If degradation occurs too rapidly, the material may lose its support before sufficient cartilage matrix is deposited; if degradation is too slow, it may hinder cell migration and extracellular matrix remodeling [235]. Within the broader therapeutic framework of sports injuries, these hydrogels not only act as fillers and structural supports for defect sites but also serve as active regulators at the molecular and cellular levels, offering promising prospects for functional cartilage reconstruction [236]. Thus, the design of protein-based hydrogels for cartilage injury repair should follow a structure–property–function logic, in which material composition, network architecture, mechanical behavior, degradation profile, and release kinetics are coordinated to promote durable and functional cartilage regeneration [149].

#### 5.1.1. Regulation of Cellular Behavior via Mechanical Properties and Scaffold Architecture

In the repair of cartilage injuries, establishing an appropriate mechanical microenvironment is critical for promoting chondrocyte proliferation, differentiation, and matrix synthesis [45]. Articular cartilage is inherently subjected to complex mechanical loading, and its cellular function and tissue homeostasis are highly dependent on mechanical stimulation signals [237]. Moderate compressive, tensile, and shear stresses not only activate mechanosensitive pathways in chondrocytes—regulating the expression of growth factors and extracellular matrix proteins—but also suppress inflammatory responses and apoptosis, thereby creating favorable conditions for tissue regeneration [238]. Consequently, recreating and simulating physiological mechanical cues has become a key strategy to enhance repair outcomes and achieve functional regeneration.

**Figure 6:**
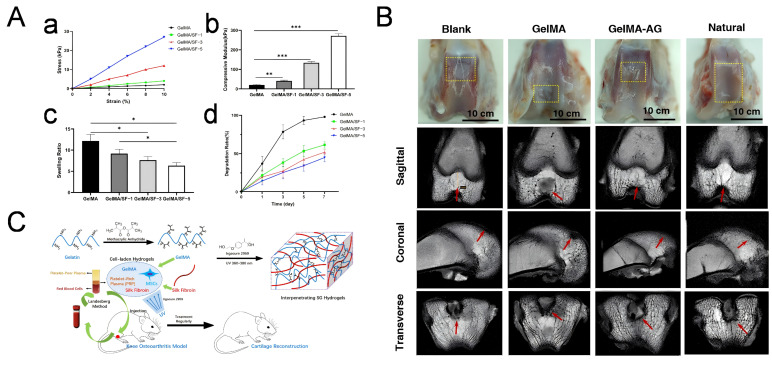
(**A**): Physical properties of GelMA/SF composite hydrogels. (**a**): The representative stress-strain curves of hydrogels with different formations; (**b**): The compressive modulus of each hydrogel (*n* = 5); (**c**): The swelling property of each hydrogel in PBS solution at room temperature for 24 h (*n* = 4); (**d**): Degradation ratio of each hydrogel in PBS solution containing 2 U/mL collagenase type l at 37 °C after 1, 3, 5, and 7 days (*n* = 4). Significant difference symbols: * = *p* < 0.05; ** = *p* < 0.01; *** = *p* < 0.001 [242]. (**B**): Macroscopic observation and magnetic resonance imaging (MRI) of cartilage defect at 12 weeks post-surgery [245]. (**C**): A schematic illustration of the preparation of MSCs-laden gelatin methacrylate/silk fibroin (GelMA/SF, SG) hydrogel with the incorporation of PRP for treating KOA to reconstruct cartilage [247].

Because of their tunable properties, protein-based hydrogels provide a versatile platform for modulating mechanical performance and optimizing the physical microenvironment for cell growth, thereby facilitating proliferation and matrix deposition [239]. Lei et al. developed a composite hydrogel based on gelatin methacrylamide (GelMA) and oxidized chondroitin sulfate (OCS), in which OCS served as a functional crosslinker. The incorporation of OCS significantly enhanced the compressive modulus (up to 16.085 kPa), rendering the mechanical profile closer to native cartilage and enabling effective load-bearing under physiological stress. In a rat cartilage defect model, the GelMA/OCS hydrogel promoted robust cartilage regeneration, with newly formed tissue integrating well with host cartilage and outperforming controls. To further strengthen both mechanical and biological properties, researchers have adopted multicomponent strategies [240] Murphy et al. engineered UV-crosslinkable and 3D-printable GelMA-based hydrogels incorporating chondroitin sulfate (CS) and hyaluronic acid (HA). The compressive modulus of these GelMA/glycosaminoglycan hybrid hydrogels could be tuned within the range of 36–93 kPa, suggesting that their stiffness could be adjusted to meet different cartilage repair requirements. Moreover, cell experiments demonstrated that HA- and HA/CS-containing porous constructs supported higher DNA content and metabolic activity by day 7; in particular, freeze-dried porous scaffolds showed approximately 30–57% higher DNA content than their corresponding non-freeze-dried counterparts. These findings indicate that HA and CS contribute not only to mechanical reinforcement but also to a more favorable cellular microenvironment. Beyond compositional tuning, introducing specific proteins has also been explored to enhance hydrogel mechanics and functionality [241]. Zheng et al. incorporated silk fibroin (SF) into a GelMA-based interpenetrating polymer network (IPN) hydrogel. GelMA provided favorable cell adhesion and compatibility, while SF markedly reinforced the mechanical properties, achieving a compressive modulus of 300 kPa and reducing degradation rates, thus offering more durable support for cartilage regeneration (Figure 6A). In vivo experiments using a rat knee cartilage defect model confirmed that GelMA/SF hydrogels loaded with bone marrow-derived mesenchymal stem cells (BMSCs) significantly promoted cartilage repair, resulting in smooth surfaces and abundant matrix deposition [242].

Beyond enhancing intrinsic material mechanics, designing protein-based hydrogels as scaffolds tailored to tissue requirements is another pivotal approach. Scaffold performance can be modulated through pore size and architecture to provide favorable niches for cellular growth [243]. For example, Liu fabricated gelatin-based hydrogel scaffolds with uniform pore sizes (100 µm and 160 µm) and highly interconnected networks. The scaffold with 160 μm pores provided a more favorable microenvironment for chondrocyte matrix production. Specifically, the GAG/DNA ratio increased from approximately 0.2 μg on day 1 to approximately 1.1 μg on day 21 in the 100 μm scaffold, whereas the 160 μm scaffold reached approximately 2.0 μg on day 21. Under dynamic culture for up to 28 days, type II collagen expression gradually increased, while Sox9 and aggrecan expression were maintained at later time points, indicating that optimized pore architecture helped preserve the chondrocyte phenotype and promote extracellular matrix secretion [244]. Yang et al. developed gelatin hydrogel scaffolds capable of releasing glutamine during degradation. The released glutamine served as a nutrient source for chondrocytes, supporting energy metabolism and facilitating cartilage repair. At 12 weeks post-surgery, macroscopic evaluation and MRI analysis of the cartilage defects further confirmed the repair-promoting effect of this hydrogel system (Figure 6B) [245]. Similarly, Pei developed a 3D-bioprinted GelMA scaffold seeded with mesenchymal stem cells (MSCs), in which upregulation of miRNA-410 enhanced cell migration, proliferation, and chondrogenic differentiation. In a rabbit cartilage defect model, the GelMA-MSC scaffold with miRNA-410 overexpression showed markedly improved repair at both 6 and 12 weeks, with regenerated tissue closely resembling native cartilage [246]. Furthermore, Chen designed a silk fibroin–GelMA IPN hydrogel (SG hydrogel) combined with platelet-rich plasma (PRP) to harness growth factors for enhancing MSC proliferation and chondrogenic differentiation (Figure 6C). After 8 weeks, histological evaluation demonstrated that the MSCs-laden SG hydrogel + PRP group achieved the most favorable cartilage reconstruction, with a Mankin score of 3.0 ± 0.91, which was markedly lower than that of the control group (12.5 ± 0.50), SG hydrogel group (6.5 ± 0.46), and SG hydrogel + PRP group (5.5 ± 0.72). The regenerated cartilage in this group showed a smoother surface and more orderly chondrocyte arrangement, indicating that the combination of MSCs, SF/GelMA scaffold architecture, and PRP-derived bioactive cues can synergistically enhance cartilage regeneration [247].

#### 5.1.2. Modulation of the Inflammatory Microenvironment

During the acute inflammatory phase of cartilage injury, the avascular and aneural nature of cartilage leads to limited blood supply and delayed infiltration of inflammatory cells such as macrophages [248]. However, the prolonged retention of pro-inflammatory cytokines (e.g., IL-1β, TNF-α, IL-6) activates catabolic pathways including NF-κB and MAPK, resulting in excessive expression of matrix metalloproteinases (MMPs) and ADAMTS family enzymes that degrade collagen and proteoglycans [249]. Moreover, the persistent inflammatory milieu suppresses chondrocyte synthesis of type II collagen and aggrecan, while inducing apoptosis and fibrotic transformation, thereby generating mechanically inferior fibrocartilage [250]. Consequently, effective regulation of the inflammatory component is essential in cartilage repair to promote type II collagen and aggrecan synthesis.

**Figure 7:**
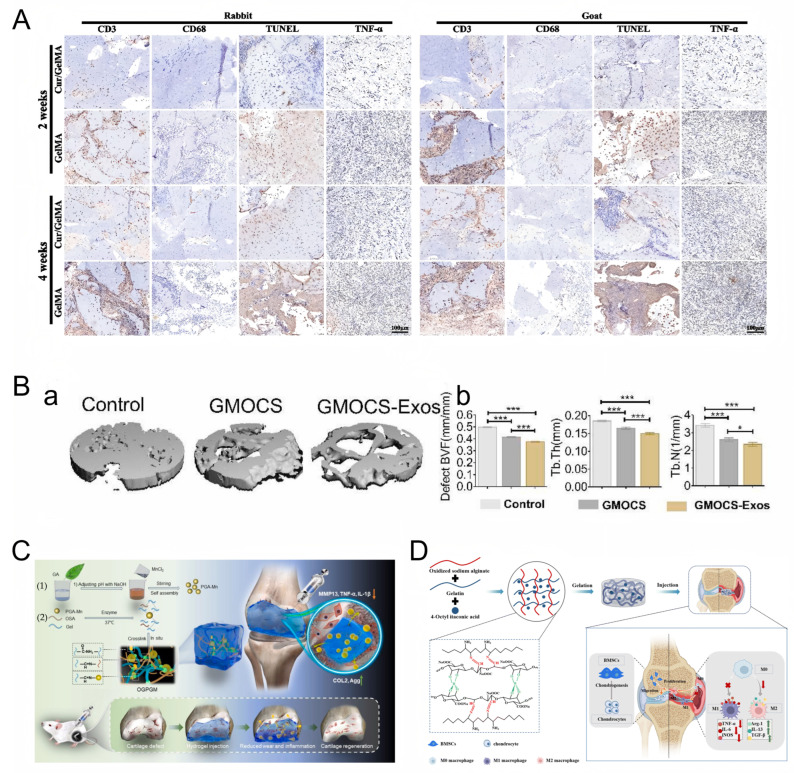
(**A**): Inflammatory reactions in rabbits and goats at 2 and 4 weeks were assessed using immunohistochemical staining (CD3, CD68, TUNEL, TNF-α) of subcutaneously implanted samples from the Cur/GelMA and GelMA groups in rabbits [252]. (**B**): (**a**): Micro-CT images of bone bridge formation in the defect; (**b**): Quantitative measurement of bone bridge formation at the defect site (*n* = 3, * *p* < 0.05 and *** *p* < 0.001) [253]. (**C**): Preparation and action process of nanocomposite hydrogels [254]. (**D**): Flowchart of hydrogel fabrication and hydrogel treatment for cartilage defects by regulating inflammation through early macrophage polarization [255].

To mitigate inflammation in cartilage lesions, anti-inflammatory drugs can be incorporated into hydrogel matrices, leveraging the excellent delivery performance of protein-based hydrogels to achieve sustained local effects [251]. Multiple strategies have been developed. For instance, Li et al. designed an anti-inflammatory Cur/GelMA hydrogel, where curcumin (Cur), a phytochemical with intrinsic anti-inflammatory properties, was encapsulated into gelatin methacrylate (GelMA) hydrogels. Continuous release of Cur effectively suppressed pro-inflammatory cytokine expression (IL-1β, TNF-α, IL-6, COX-2), reduced inflammatory cell (CD3, CD68) infiltration, and alleviated tissue damage, thereby protecting chondrocytes. In vivo studies demonstrated that Cur/GelMA hydrogels significantly promoted cartilage regeneration in rabbit and goat models, with superior regenerative outcomes and attenuated inflammation compared to controls at both 2 and 4 weeks (Figure 7A) [252]. Similarly, Xiao et al. developed an injectable OSA/GEL hydrogel loaded with 4-octyl itaconate (4-OI) for inflammatory microenvironment regulation and tissue repair. Mechanistically, 4-OI suppressed M1 macrophage polarization and pro-inflammatory cytokine secretion while promoting M2 macrophage polarization and anti-inflammatory factor expression, thereby restoring local immune balance. Owing to its favorable biocompatibility, biodegradability, and mechanical stability, the hydrogel enabled sustained 4-OI release and effective inhibition of inflammatory responses. Micro-CT imaging and quantitative analysis further demonstrated enhanced bone bridge formation at the defect site, supporting the repair-promoting potential of this hydrogel system (Figure 7B) [253]. In another study, Guan et al. developed an extracellular matrix-mimicking hydrogel carrying exosomes (GMOCS-Exos). By encapsulating bone marrow mesenchymal stem cell (BMSC)-derived exosomes, GMOCS-Exos hydrogel delivered anti-inflammatory mediators such as TGF-β, IL-10, and regulatory miRNAs (e.g., miR-146a, miR-181c), thereby modulating macrophage polarization (M1→M2) and inhibiting T cell activation to improve the local immune microenvironment. Meanwhile, oxidized chondroitin sulfate (OCS) was incorporated to directly supplement ECM components, enhancing chondrocyte proliferation and differentiation (Figure 7C). Both in vitro and in vivo results confirmed that GMOCS-Exos hydrogels significantly increased chondrocyte viability and proliferation, upregulated cartilage-specific genes (SOX-9, COL-2), reduced osteophyte formation, and promoted cartilage regeneration [254].

Additionally, incorporation of metal ions into hydrogels has been explored to scavenge reactive oxygen species (ROS) and alleviate inflammation. Chen et al. constructed OGPGM hydrogels by combining poly(gallic acid)-manganese (PGA-Mn) nanoparticles with OSA and gelatin (Figure 7D). PGA-Mn endowed the hydrogel with efficient reactive oxygen species-scavenging ability and improved mechanical performance through crosslinking interactions with gelatin. In material characterization, OGPGM(1:5) showed a swelling ratio of approximately 550% after PBS immersion and finally reached approximately 1200%, indicating strong water-absorption capacity. Its coefficient of friction decreased from approximately 0.03 to 0.012, suggesting enhanced lubrication, while its compressive modulus increased from 168.07 kPa to 264.89 kPa. These properties are relevant to cartilage repair because lubrication and mechanical support are essential for reducing intra-articular friction and protecting cartilage under repeated joint loading. In vitro, OGPGM effectively removed ROS from chondrocytes and reduced the expression of inflammatory factors such as TNF-α and IL-1β. In a rat osteoarthritis model, OGPGM reduced osteophyte formation and protected cartilage from wear, with the OGPGM-treated group showing the lowest OARSI score of approximately 3.33 [255].

#### 5.1.3. Promotion of Osteochondral Integration

In cartilage repair, beyond restoring the intrinsic regenerative capacity of cartilage tissue, achieving effective integration between cartilage and the underlying subchondral bone is equally crucial for functional recovery [50]. Such integration is essential for reconstructing a stable cartilage–bone interface and restoring normal joint biomechanics [256]. Given the pronounced structural and functional disparities between cartilage and bone, protein-based hydrogels—with their excellent biocompatibility, tunable mechanical properties, and capacity to mimic the native extracellular matrix (ECM)—have emerged as highly promising candidates for promoting osteochondral integration [257].

**Figure 8:**
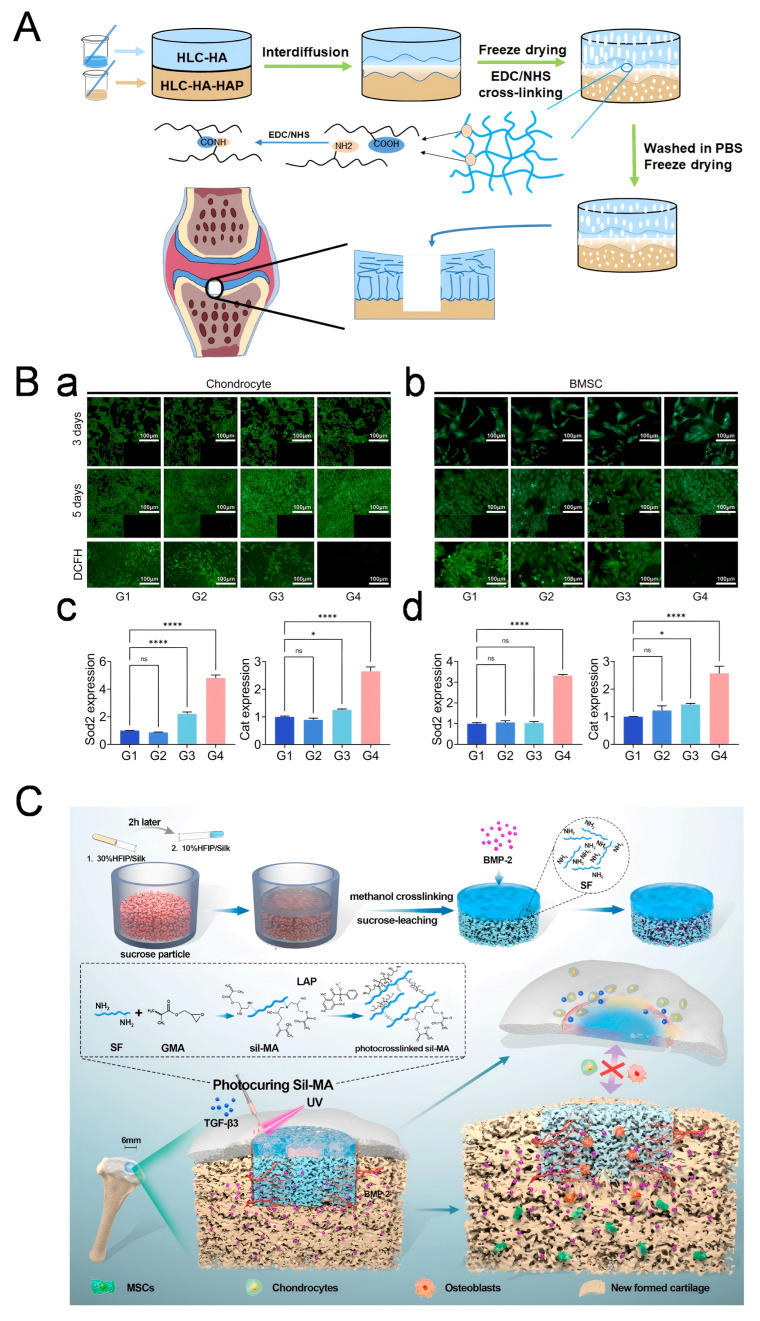
(**A**): Methodology used for preparing the bilayer scaffolds, creating HLC-HA as cartilage-like layers in combination with HLC-HA-HAP as subchondral bonelike layers [258]. (**B**): Biocompatibility and anti-inflammatory performance of the bilayer hydrogel scaffold. (**a**): Live/dead staining and ROS scavenging staining of bilayer hydrogel scaffolds in rat chondrocytes; (**b**): Live/dead staining and ROS scavenging staining of bilayer hydrogel scaffolds in rat BMSCs; (**c,d**): mRNA expression of two antioxidative genes in rat chondrocytes and BMSCs after treatment with hydrogel scaffolds (* *p* < 0.05; **** *p* < 0.0001) [259]. (**C**): Schematic illustration of the integral bilayer silk scaffold combined with Sil-MA hydrogel in osteochondral repair [262].

Current research primarily focuses on engineering bilayer scaffolds to facilitate osteochondral regeneration. For example, Liu et al. designed a biomimetic bilayer scaffold, where the upper layer consisted of human-like collagen (HLC) and hyaluronic acid (HA) to mimic the cartilage ECM, while the lower layer incorporated nano-hydroxyapatite (HAP) to simulate bone tissue (Figure 8A). In vitro studies demonstrated enhanced proliferation and adhesion of human bone marrow mesenchymal stem cells (hBMSCs) on this scaffold. In a rabbit knee osteochondral defect model, the bilayer scaffold group achieved superior outcomes in International Cartilage Repair Society (ICRS) scores, micro-CT imaging, and histological staining at 8 and 12 weeks compared to both blank controls and single-layer scaffolds, indicating favorable osteochondral healing [258]. Similarly, Hu et al. further introduced an immunoregulatory and antioxidant strategy by constructing a LiMn_2_O_4_ nanozyme-functionalized bilayer GelMA-based hydrogel scaffold. The cartilage-like layer consisted of HAMA/GelMA and LiMn_2_O_4_, while the subchondral bone-like layer was composed of alginate/GelMA, hydroxyapatite, and LiMn_2_O_4_. This design allowed the scaffold to simultaneously mimic the hierarchical osteochondral structure and regulate the oxidative inflammatory microenvironment. LiMn_2_O_4_ endowed the scaffold with SOD-like, CAT-like, and Gpx-like catalytic activities, enabling efficient ROS scavenging. In vitro, the GH@LM + GA@HLM hydrogel significantly reduced intracellular ROS in rat chondrocytes and BMSCs, upregulated antioxidant genes and proteins including SOD2 and CAT, and promoted M2 macrophage polarization by increasing CD206 expression and decreasing CD86 expression. In chondrocytes exposed to oxidative stress, GH@LM treatment downregulated MMP13 while upregulating Col II and Aggrecan, indicating protection against cartilage matrix degradation. RNA-seq analysis further confirmed the downregulation of inflammatory and matrix-degrading genes such as Mmp3 and Mmp13 and the upregulation of cartilage matrix-related genes such as Col2a1 and Col11a1. In a rat osteochondral defect model, micro-CT analysis at 6 and 12 weeks showed that BV/TV and Tb.N were significantly optimized in the GH@LM + GA@HLM group. Histological staining demonstrated complete subchondral bone and partial cartilage repair at 6 weeks, followed by integrated osteochondral repair at 12 weeks. Immunofluorescence analysis showed the highest expression of cartilage-related markers Col II and Aggrecan, as well as bone-related markers Col I and Opn, in the GH@LM + GA@HLM group (Figure 8B). This scaffold not only provided physical support but also facilitated osteochondral repair through the controlled release of bioactive factors [259]. Beyond standalone bilayer scaffolds, integration with additional hydrogel systems has been shown to further enhance repair outcomes. Wu et al. developed a composite strategy combining a bilayer silk fibroin scaffold with a photocrosslinkable silk methacrylate (Sil-MA) hydrogel. The upper layer of the silk fibroin scaffold acted as a cartilage-mimicking zone, providing a smooth surface for chondrocyte growth, while the lower porous bone layer was loaded with BMP-2 to stimulate osteogenic migration and differentiation. Meanwhile, Sil-MA hydrogel, serving as a marginal sealant, was loaded with TGF-β3 to fill interfacial gaps and guide new cartilage formation, enabling lateral tissue integration (Figure 8C). In release experiments, approximately 50% of TGF-β3 was released from the Sil-MA hydrogel after 10 days, and the cumulative release reached approximately 60% after 21 days, indicating sustained delivery of chondrogenic cues. In migration assays, chondrocyte migration was observed as early as 6 h in the TGF-β3/Sil-MA group and became more evident at 12 h. In vivo, the BMP-2-loaded bilayer scaffold combined with TGF-β3-loaded Sil-MA hydrogel achieved improved osteochondral regeneration and superior lateral integration, as new cartilage grew from the surrounding native cartilage toward the defect and gradually replaced the degraded cartilage layer.

Despite these promising findings, the therapeutic value of protein-based hydrogels for cartilage repair should be interpreted cautiously. Many current studies remain based on in vitro experiments or small-animal defect models [260], and improved histological appearance does not necessarily indicate restoration of native hyaline cartilage mechanics. In sports-related cartilage injuries, the repaired tissue must withstand repeated compression, shear stress, and joint motion. However, many protein-based hydrogels still have difficulty reproducing the zonal architecture, compressive modulus, lubrication behavior, and long-term load-bearing capacity of native cartilage. Future studies should therefore compare hydrogel-based cartilage [149] repair not only with untreated controls, but also with clinically used approaches such as microfracture, hyaluronic acid injection, PRP therapy, and cell-based cartilage repair [261].

### 5.2. Muscle Injury Repair

Protein-based hydrogels have shown considerable potential in the repair of sports-related muscle injuries because they can provide both structural support and a biologically active microenvironment [263]. Unlike cartilage or bone tissue, damaged skeletal muscle requires not only the restoration of structural continuity but also the reconstruction of its highly organized contractile function and mechanical coordination [264]. Therefore, the therapeutic performance of hydrogels in muscle repair depends not only on general biocompatibility, but also on whether their elasticity, porosity, degradation behavior, and microstructural organization can support functional myogenesis [265].

Owing to their tunable viscoelasticity and excellent biomimetic properties, protein-based hydrogels can provide a microenvironment that closely resembles native muscle tissue to support myofiber regeneration after injury. For muscle repair, key structure–property requirements include elasticity, injectable defect filling, aligned microarchitecture, controlled degradation, and vascular–neural permissiveness. These properties determine whether the hydrogel can support myoblast migration, myotube alignment, angiogenesis, reinnervation, and integration with host contractile tissue [266]. On the one hand, their highly hydrated and porous network effectively relieves localized stress concentrations, prevents excessive scar tissue deposition, and thereby reduces the risk of functional contracture and secondary injury. On the other hand, their biomimetic support promotes satellite cell adhesion, proliferation, and myogenic differentiation, facilitating the orderly alignment and functional integration of newly formed myofibers [267]. However, these biological effects are closely related to hydrogel structure. A network that is too dense may restrict cell migration, vascular ingrowth, and nutrient diffusion, whereas a network that is too loose may degrade prematurely or fail to maintain defect stability during muscle contraction [268].

In addition, protein-based hydrogels exhibit unique load-adaptive properties, maintaining stable mechanical performance under dynamic stresses such as running, jumping, and stretching, which aligns with the clinical principle of “progressive loading during rehabilitation [269].” This mechanical adaptability is particularly important because injured muscle is repeatedly exposed to contraction, stretching, and rehabilitation-related loading. If the hydrogel is too stiff, it may interfere with muscle deformation and mechanotransduction; if it is too soft or mechanically unstable, it may fail to transmit load, maintain defect filling, or support organized myofiber regeneration [264]. Therefore, hydrogels for muscle injury repair should be evaluated not only by cell viability or short-term anti-inflammatory effects, but also by their ability to guide aligned myofiber formation, vascular–nerve integration, force generation, and functional recovery under dynamic loading conditions [270].

More importantly, hydrogels can serve as localized therapeutic platforms by delivering drugs, cells, or growth factors, enabling precise regulation of inflammatory responses and promoting angiogenesis to support functional regeneration at multiple levels [271]. The timing and duration of bioactive release are also critical [272]. Early anti-inflammatory release may help control excessive inflammation and fibrosis, whereas later-stage delivery of myogenic or angiogenic cues may better support satellite cell activation, vascular remodeling, and maturation of newly formed muscle fibers [273]. Therefore, the release behavior of muscle-oriented hydrogels should be coordinated with the temporal sequence of inflammation resolution, cell recruitment, myofiber formation, and tissue remodeling. Therefore, in the context of exercise-induced muscle injury repair, protein-based hydrogels act not only as physical scaffolds but also as regulators of the regenerative microenvironment, accelerators of functional restoration, and enablers of shortened rehabilitation timelines. Overall, the design of protein-based hydrogels for muscle injury repair should follow a structure–property–function logic, in which elasticity, pore architecture, degradation rate, alignment cues, and release kinetics are coordinated to promote not only histological regeneration, but also functional muscle recovery [266].

#### 5.2.1. Regulation of Myogenic Cell Proliferation and Differentiation

Sufficient proliferation of myogenic precursor cells, particularly satellite cells, is a decisive step in the functional repair of muscle injuries. These cells provide the essential source of myoblasts required for new myofiber formation and structural-functional recovery [274]. Insufficient proliferation leads to impaired regeneration, making the injury site prone to infiltration by fibrotic or adipose tissue with limited functionality, thereby severely compromising muscle strength and the restoration of motor capacity [275]. Therefore, effectively promoting the proliferation of myogenic precursor cells is the primary prerequisite for optimizing muscle regeneration. Protein-based hydrogels, with their favorable physicochemical and biological properties, offer an effective strategy to stimulate myogenic cell proliferation and differentiation, thereby accelerating functional recovery [151].

**Figure 9:**
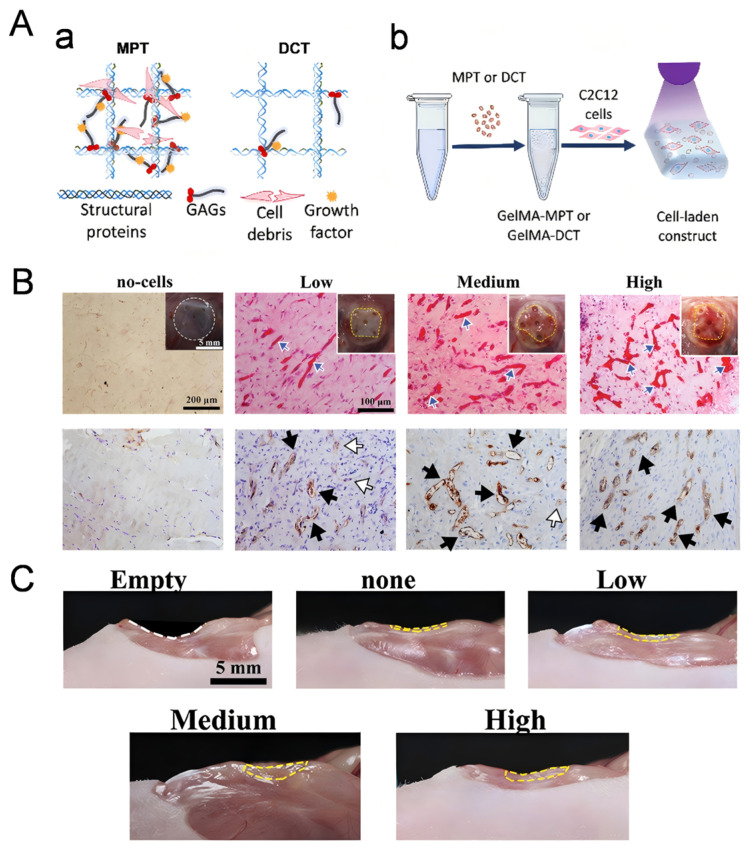
(**A**): (**a**): Schematic representation of structural and composition differences between MPT and decellularized tissue (DCT) powders; (**b**): Preparation of the GelMA-MPT or GelMA-DCT hydrogels for producing cell-laden constructs [276]. (**B**): On the 7th day after subcutaneous implantation in mice, collagen hydrogels with varying cell densities—from cell-free to low, medium, and high—effectively modulated the density of blood vessels formed within the hydrogels. Macroscopic and cross-sectional H&E staining images revealed the formation of perfused blood vessels (blue arrows) in cell-laden hydrogels. Immunohistochemical staining further identified human vessels (hCD31-positive, brown, solid black arrows) and mouse host vessels (hCD31-negative, hollow arrows) within the hydrogels. (**C**): Macroscopic lateral-view images of the defect site after 8 weeks show that increasing cell density in three-layer hydrogel implants significantly enhances muscle repair in mice, displaying results for the empty, cell-free (none), low, medium, and high groups [277].

Tavares-Negrete et al. investigated the incorporation of minimally processed tissue (MPT) powder derived from skeletal muscle into gelatin methacryloyl (GelMA) hydrogels to form dense skeletal muscle-like constructs (Figure 9A). MPT was prepared by freeze-drying, grinding, and sieving skeletal muscle tissue, thereby preserving bioactive components such as DNA, proteins, and glycosaminoglycans. Compared with pristine GelMA, GelMA-MPT hydrogels provided a more muscle-specific biochemical microenvironment and significantly improved C2C12 cell proliferation and alignment. Importantly, GelMA-MPT constructs maintained their three-dimensional structural integrity for up to 28 days, whereas comparable unmodified GelMA constructs generally maintained their structure for 14 days or less. These results indicate that MPT supplementation not only enhances cellular organization but also improves the long-term stability of engineered muscle-like tissues [276]. Moreover, these hydrogels exhibited excellent printability in extrusion-based bioprinting, enabling the fabrication of high-fidelity constructs and offering a simple, cost-effective approach for tissue engineering and regenerative medicine. In another study, Wei et al. developed collagen-based hydrogel implants with prevascularized networks of varying densities by modulating the ratio of human umbilical vein endothelial cells (HUVECs) to mesenchymal stem cells (MSCs). Collagen hydrogels with higher vascular densities (0–145 vessels/mm^2^) were able to integrate effectively with host vasculature upon implantation, enhancing neovascularization and providing sufficient nutrients and oxygen to support muscle cell proliferation and regeneration (Figure 9B). Importantly, increased vascular density also promoted host neuronal ingrowth and migration. Comparative experiments further demonstrated that complete filling of muscle defects with stacked multilayer hydrogels (1 mm each) led to a repair rate of 91% at 8 weeks in the high-density group, approaching that of native muscle tissue (Figure 9C). Behavioral testing confirmed that mice treated with high-density vascularized hydrogels regained muscle strength and functional performance, comparable to healthy controls. These findings highlight that prevascularized hydrogels can rapidly integrate with host vascular and neural networks, thereby providing robust support for muscle regeneration [277].

Beyond merely stimulating myogenic proliferation, directing the alignment of myofibers is equally critical, as the fixed orientation of myofiber bundles underpins their contractile functionality. Thus, a key scientific challenge in muscle regeneration lies in guiding newly formed myofibers to grow, align, and proliferate along specific orientations consistent with native muscle architecture. Protein-based hydrogels offer promising platforms to induce such anisotropic regeneration through mechanical and biochemical cues. For instance, Egorova developed a fibrous hydrogel (MyoColl) composed of gelatin, oxidized sodium alginate (OSA), and type I collagen, crosslinked via Schiff base reactions to mimic the fibrillar architecture of native extracellular matrix (ECM). MyoColl promoted muscle regeneration through mechanotransduction, wherein uniaxial stretching converted mechanical signals into biochemical cues, thereby enhancing myotube formation and alignment [278]. In addition to mechanical guidance, anisotropy can be achieved through magnetic or conductive modifications. Shi designed a magnetically induced anisotropic conductive in situ hydrogel based on hyaluronic acid methacrylate (HAMA) and GelMA, incorporating polydopamine (PDA)-coated carbon nanotubes (CNTs) and Fe_3_O_4_ nanoparticles (PFeCNT). This hydrogel formed anisotropic structures under an external magnetic field, mimicking natural muscle fiber alignment, promoting directional myogenic growth and differentiation. Furthermore, its conductivity facilitated intercellular electrical signaling, accelerating muscle maturation. The hydrogel was biodegradable, minimizing long-term inflammatory risks, while the addition of tryptophan (Trp) further enhanced myogenic differentiation. In a tibialis anterior defect model in Sprague–Dawley rats, this multifunctional hydrogel markedly promoted the formation of new muscle fibers and capillaries, resulting in significantly improved muscle repair outcomes [279].

#### 5.2.2. Regulation of the Inflammatory Microenvironment

The repair process following muscle injury is highly dependent on the inflammatory response, yet its precise regulation is crucial. Acute inflammation facilitates debris clearance and initiates regeneration. However, excessive or persistent inflammation becomes detrimental, as the production of reactive oxygen species (ROS) and reactive nitrogen species (RNS) during the inflammatory cascade induces oxidative stress, leading to secondary tissue damage, impaired myofiber regeneration, and pathological fibrosis. This creates a sustained inflammation–oxidative stress cycle that ultimately delays functional recovery [57,280]. Therefore, accurately modulating inflammation—particularly promoting timely resolution and attenuating oxidative stress—is a key strategy for optimizing muscle repair. Breaking this cycle through ROS scavenging represents an upstream anti-inflammatory intervention that effectively mitigates both inflammatory responses and associated tissue damage.

**Figure 10:**
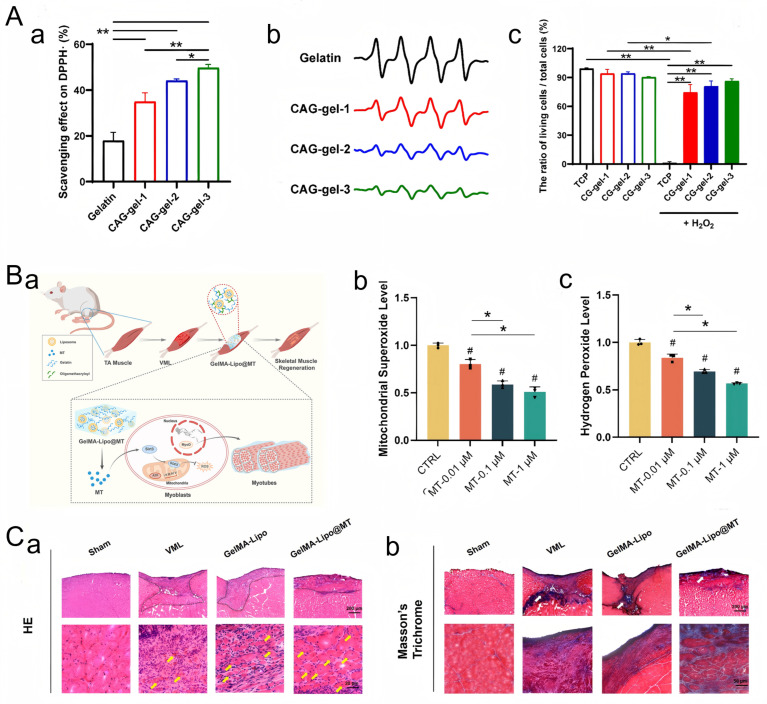
(**A**): (**a**): DPPH radical scavenging ability of CAG-gels, *n* = 3; (**b**): Hydroxyl radical (-OH) scavenging ability of CAG-gels measured by ESR spectroscopy; (**c**): Quantitative analysis of BMSC viability with or without the treatment of 100 μM H_2_O_2_, *n* = 3. * *p* < 0.05, ** *p* < 0.01 [282]. (**B**): (**a**): Melatonin enhances myotube formation via SIRT3, boosting mitochondrial function and antioxidant activity by activating the SIRT3-SOD2 axis. For VML repair, melatonin-loaded liposomes (GelMA-Lipo@MT) implanted in muscle defects promoted regeneration, angiogenesis, and reduced fibrosis; (**b**): Quantification of mitochondrial superoxide in melatonin-treated cells, *n* = 3. (**c**): Quantification of intracellular hydrogen peroxide in melatonin-treated cells using Amplex™ Red probe (Molecular Probes, Eugene, OR, USA), *n* = 3, ^#^ *p* < 0.05. (**C**): Histological evaluation of skeletal muscle regeneration of VML injury at 4 weeks post-surgery. GelMA-Lipo and GelMA-Lipo@MT hydrogels were implanted into the injury site, while the VML group was left untreated. (**a**): Representative hematoxylin and eosin (H&E) staining of the newly regenerated myofibers with central nuclei (the yellow arrows); (**b**): Representative Masson’s trichrome staining of collagen deposition indicates the fibrotic tissue (the white arrows) [283].

In the context of muscle repair, controlling oxidative stress is central to controlling inflammation [281]. One promising approach involves the use of natural bioactive extracts with antioxidant properties. Hu developed an injectable antioxidant hydrogel (CAG-gel) formed via laccase-catalyzed crosslinking of caffeic acid-modified gelatin, which exhibited excellent antioxidant activity and mechanical stability. CAG-gel effectively scavenged ROS, protecting stem cells from oxidative injury while preserving their stemness and multilineage differentiation potential (Figure 10A). Moreover, CAG-gel significantly improved stem cell retention at the injury site, promoted their differentiation into myofibers, and enhanced muscle tissue regeneration. Experimental results demonstrated that stem cells encapsulated in CAG-gel formed denser, more uniform myofibers with significantly increased cross-sectional areas. In addition, CAG-gel promoted angiogenesis, improved local perfusion, and attenuated inflammation, collectively contributing to enhanced tissue repair. This hydrogel platform thus provides a new tool for advancing stem cell therapies in muscle injury repair [282]. Another strategy is the incorporation of melatonin into hydrogels to mitigate oxidative stress. Melatonin has been shown to significantly enhance the differentiation of C2C12 myoblasts and promote myogenesis by upregulating the expression of myogenic markers (e.g., *MyoD*, *Myog*, and *MHC*) in a dose-dependent manner. It also protects myoblasts by improving mitochondrial energy metabolism and activating mitochondrial antioxidant enzymes such as SOD2. Notably, silencing the Sirt3 gene completely abolished melatonin’s protective effects on mitochondrial function. Building upon these findings, Ge and colleagues developed a composite system combining melatonin with gelatin methacryloyl (GelMA) hydrogels to promote vascularized skeletal muscle regeneration after volumetric muscle loss (VML). To enable sustained melatonin release, liposome-encapsulated melatonin was integrated into GelMA hydrogels (GelMA-Lipo@MT). In vitro, melatonin treatment significantly enhanced ATP production and mitochondrial membrane potential (MMP) in C2C12 cells, while reducing ROS levels (Figure 10B). In vivo, implantation of GelMA-Lipo@MT into a rat tibialis anterior VML model demonstrated robust muscle regeneration and neovascularization at 4 weeks. Histological analyses revealed increased formation of newly regenerated myofibers and markedly reduced collagen deposition (Figure 10C). Immunofluorescence staining further confirmed upregulated expression of MHC, SIRT3, SOD2, and CD31, indicating enhanced myofiber and vascular formation [283].

For muscle injury repair, protein-based hydrogels provide favorable biomimetic microenvironments, but their ability to restore functional muscle tissue remains limited [263]. Skeletal muscle regeneration requires not only myogenic cell proliferation, but also aligned myofiber formation, vascularization, reinnervation, and integration with host contractile tissue [266,284]. Many hydrogel systems can improve cell survival or reduce inflammation, but evidence for restoring contractile strength, fatigue resistance, neuromuscular connectivity, and sport-specific functional performance remains insufficient. Future muscle repair studies should move beyond short-term histological regeneration and include quantitative assessments of force generation, fiber alignment, vascular–nerve integration, scar formation, and functional recovery under dynamic loading conditions [263,284,285].

### 5.3. Tendon Repair

Protein-based hydrogels have shown considerable potential in the repair of sports-related tendon injuries because they can provide both bioactive support and a tunable extracellular matrix-like environment [242]. Unlike other tissues, tendons simultaneously serve as high-tension force transmitters and regulators of fine motor control. Therefore, effective repair after injury requires not only restoration of tissue continuity but also reestablishment of highly organized collagen alignment and load-bearing adaptability [286]. For tendon repair, the key structure–property requirements of hydrogels include tensile strength, anisotropic architecture, fatigue resistance, adhesive retention, and degradation synchronized with collagen remodeling. These properties determine whether hydrogels can support aligned collagen deposition, reduce adhesion, regulate inflammation, and restore force transmission.

Owing to their tunable viscoelasticity and biomimetic microenvironment, protein-based hydrogels can form a matrix-like framework at the injury site, providing mechanical cues for tendon cell adhesion and alignment. This facilitates the ordered reconstruction of nascent collagen fibers along the direction of tensile loading, thereby avoiding scar-mediated repair and functional imbalance [287]. However, this effect is strongly dependent on hydrogel network structure [288]. A randomly organized hydrogel network may support cell survival but may not adequately guide the highly aligned collagen architecture required for tendon force transmission. In contrast, aligned, fiber-reinforced, or anisotropic hydrogel structures may better reproduce the directional mechanical cues of native tendon and promote more organized tenogenic remodeling [288,289].

Moreover, the high water content and porous architecture of hydrogels not only enable efficient exchange of nutrients and signaling molecules but also serve as delivery platforms for anti-inflammatory factors, angiogenic agents, and stem cells, thus achieving dual regulation of inflammation suppression and regeneration promotion [290]. The pore size, interconnectivity, and degradation rate of the hydrogel further influence tenocyte migration, vascular ingrowth, collagen deposition, and matrix maturation [291]. If the hydrogel degrades too rapidly, it may lose mechanical support before sufficient collagen remodeling occurs; if it degrades too slowly, it may restrict cell infiltration, delay matrix remodeling, or increase the risk of adhesion formation [292]. Therefore, the degradation profile of tendon-oriented hydrogels should be matched with the temporal sequence of inflammation resolution, collagen synthesis, fiber alignment, and mechanical strengthening.

Particularly in the context of sports rehabilitation, protein hydrogels can be mechanically tuned to match dynamic loading conditions, supporting early weight-bearing and functional training, reducing the risk of re-rupture, and shortening recovery time. Nevertheless, this claim should be interpreted cautiously because many protein-based hydrogels remain mechanically weaker than native tendon tissue and may not withstand repetitive tensile loading during early rehabilitation [292]. Therefore, tendon hydrogel systems should be evaluated not only by short-term cellular or histological outcomes, but also by tensile strength, fatigue resistance, interface integration, gliding function, adhesion prevention, and mechanical performance under cyclic loading [293]. Consequently, protein-based hydrogels in tendon repair function not only as structural substitutes but also as regulators of the microenvironment and mechanical response, paving the way for functional regeneration and restoration of athletic performance. Overall, the design of protein-based hydrogels for tendon repair should follow a structure–property–function logic, in which molecular composition, network alignment, mechanical durability, degradation behavior, and bioactive release are coordinated to support organized collagen remodeling and functional tendon recovery [292].

**Figure 11:**
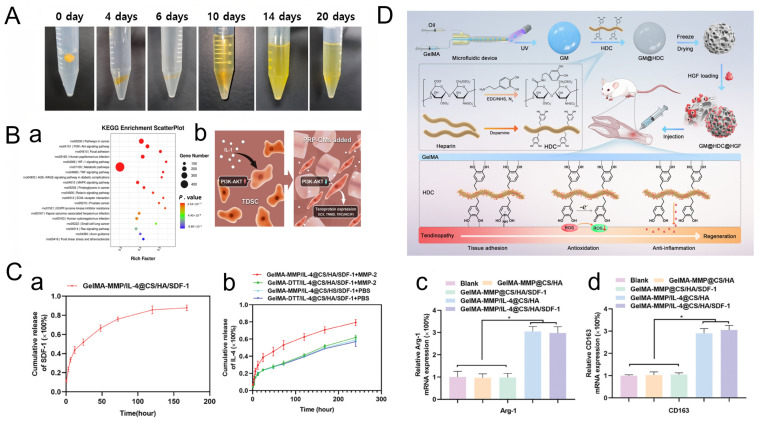
(**A**): Analysis of the growth factor release from the hydrogel, including photographs of the growth factor-loaded hydrogel at different time points [294]. (**B**): (**a**): Significantly enriched KEGG pathway terms; (**b**): PRP-GM promotes tendon repair by downregulating the PI3K-AKT pathway [295]. (**C**): (**a**): In vitro cumulative release kinetics of SDF-1 from GelMA-MMP/IL-4@CS/HA/SDF-1 micro-hydrogels; (**b**): In vitro cumulative release kinetics of IL-4 from GelMA-MMP/IL-4@CS/HA/SDF-1 micro-hydrogels in response to MMP-2 enzyme; (**c**): Relative Arg-1 gene expression; (**d**): Relative CD163 gene expression. The data were normalized to the blank group (*n* = 3). * *p* < 0.05 [296]. (**D**): Fabrication route of GM@HDC@HGF and its properties of tissue adhesion, antioxidation and anti-inflammation for the treatment of tendinopathy [297].

One major strategy is employing hydrogels as reservoirs for bioactive factors, enabling the sustained delivery of key proliferative growth factors such as FGF2, IGF-1, and PDGF. For instance, Song developed a photocrosslinked gelatin hydrogel stabilized by covalent dityrosine bonds, which allowed the co-delivery and protection of basic fibroblast growth factor (bFGF) and bone morphogenetic protein-12 (BMP-12). This system enabled the prolonged release of growth factors, thereby extending their therapeutic action and overcoming the short half-life associated with free growth factors (Figure 11A) [294]. Beyond promoting cell proliferation, certain growth factors also modulate inflammation, creating a more favorable microenvironment for tendon regeneration. For example, Li designed 3D-printed hydrogel microparticles based on gelatin methacryloyl (GelMA), incorporating platelet-derived growth factors (such as HGF, VEGF, TGF-β, and FGF from platelet-rich plasma, PRP) that promoted tendon stem/progenitor cell (TDSC) differentiation, suppressed inflammatory responses, and downregulated the PI3K-AKT pathway, thereby facilitating tendon healing. The tunable physicochemical properties of GelMA allowed customization for cell migration and differentiation, while its degradability ensured gradual clearance in vivo, leaving space for new tissue formation. Building upon this, a PRP-TDSC-GM system (GelMA microparticles loaded with PRP and TDSCs) effectively preserved the physiological activity of PRP, promoted TDSC migration and differentiation into mature tenocytes, and inhibited PI3K-AKT signaling, collectively reducing inflammation and accelerating tendon regeneration (Figure 11B) [295]. Experimental evidence confirmed structural and functional improvements, including enhanced mechanical strength of repaired tendons and improved collagen fiber alignment and diameter. In another approach, Chen designed a core–shell hydrogel system in which the core consisted of GelMA crosslinked with an MMP-2-sensitive peptide and loaded with interleukin-4 (IL-4), while the shell was constructed from chitosan, hyaluronic acid, and stromal cell-derived factor-1 (SDF-1) via layer-by-layer electrostatic deposition. This microhydrogel system achieved sustained release of SDF-1 and MMP-2-responsive release of IL-4, thereby recruiting mesenchymal stem cells (MSCs) and promoting M2 macrophage polarization. Such immunomodulation reduced inflammation and enhanced endogenous expression of transforming growth factor-β3 (TGF-β3), ultimately supporting fibrocartilage formation at the tendon–bone interface (Figure 11C). In vivo studies confirmed that this microhydrogel significantly improved tendon–bone integration, increased maximum tensile load and stress, and promoted the formation of organized collagen fibers and fibrocartilaginous entheses [296].

Furthermore, combined strategies integrating growth factors with antioxidant therapeutics have been explored to attenuate oxidative stress and enhance tendon regeneration. Han developed injectable hydrogel microspheres (GM@HDC@HGF) by integrating heparin–dopamine conjugates (HDC), which confer antioxidant and adhesive properties, with hepatocyte growth factor (HGF), known for its anti-inflammatory and matrix-modulating functions. The catechol groups of HDC provided strong adhesion, ensuring prolonged retention at the injury site and sustained therapeutic activity (Figure 11D). The anti-inflammatory and matrix-protective effects of GM@HDC@HGF were also confirmed in vitro and in vivo. In LPS-induced tenocytes, GM@HDC@HGF downregulated pro-inflammatory factors including iNOS, IL-1β, TNF-α, and IL-6, reduced the expression of matrix-degrading enzymes MMP-3 and MMP-13, and improved the abnormal COL-3/COL-1 balance associated with tendon degeneration. In a rat Achilles tendinopathy model, functionalized microspheres reduced oxidative stress and inflammatory infiltration at 1 week after administration. Serum levels of IL-1β, TNF-α, and IL-6 were decreased by approximately 50% in the GM@HDC and GM@HDC@HGF groups compared with the tendinopathy and GM groups. After 4 weeks of treatment, GM@HDC@HGF produced the most evident repair effect, as shown by a lower Bonar score, more organized collagen fiber arrangement, increased COL-1 expression, reduced abnormal COL-3 deposition, and improved extracellular matrix remodeling. These results suggest that injectable GelMA-based microspheres can treat tendinopathy by simultaneously inhibiting oxidative stress, reducing inflammation, and restoring tendon matrix homeostasis [297].

In tendon and ligament repair, the major challenge is not only biological healing but also restoration of anisotropic collagen organization and tensile mechanical strength [298]. Although protein-based hydrogels can deliver bioactive factors, reduce adhesion, and provide a cell-supportive microenvironment [293,294], many systems remain mechanically weaker than native tendon or ligament tissues and may not withstand early rehabilitation loading [299]. Moreover, randomly organized hydrogel networks may fail to reproduce the highly aligned collagen architecture required for efficient force transmission. Future hydrogel designs for tendon and ligament injuries should therefore emphasize aligned or fiber-reinforced structures, fatigue resistance, controlled degradation, interface integration, and comparison with conventional treatments such as eccentric training, PRP injection, suture repair, and graft reconstruction.

### 5.4. Fracture Repair

Protein-based hydrogels have shown promising potential as supportive biomaterial platforms in the repair of sports-related fractures [299]. Sports-induced fractures are often accompanied by complex mechanical environments and high-intensity loading. Therefore, repair strategies must not only stimulate bone regeneration but also restore the ability of bone tissue to adapt to dynamic stresses [300,301]. For bone repair, protein-based hydrogels should not be considered only as defect fillers or bioactive carriers. Their therapeutic performance depends on whether their network stiffness, degradation behavior, mineralization capacity, angiogenic support, and compatibility with mechanical fixation can match the biological and mechanical requirements of fracture healing [299].

With their highly tunable mechanical properties and biomimetic microenvironment, protein-based hydrogels can form stable three-dimensional scaffolds at fracture sites, providing favorable adhesion and differentiation conditions for osteoblasts and mesenchymal stem cells (MSCs) [302]. This, in turn, promotes the deposition and alignment of new bone matrix. However, bone repair differs from soft-tissue repair because it requires not only cell adhesion and matrix deposition, but also vascular invasion, mineral deposition, mechanical stability, and long-term remodeling under load [303]. Therefore, bone-oriented hydrogels often need to be combined with osteoinductive factors, mineral components, bioactive ions, ceramic particles, or fixation systems to provide sufficient osteoconductive and mechanical support [303,304].

Compared with conventional bone fillers, their adjustable stiffness and degradability allow mechanical adaptability throughout different healing stages, thereby reducing the risk of secondary injury and improving biomechanical integration [305]. The balance between stiffness and degradability is particularly important [190]. If the hydrogel is too soft or degrades too rapidly, it may fail to maintain local stability or retain osteogenic signals during the early repair phase. Conversely, if the hydrogel is excessively stable or degrades too slowly, it may restrict new bone ingrowth, delay remodeling, or interfere with the transition from soft callus to mineralized bone. Thus, the degradation profile of bone-oriented hydrogels should be coordinated with inflammation resolution, angiogenesis, osteogenic differentiation, mineralization, and mechanical remodeling [190,306].

Moreover, their porous and hydrated architecture enables sustained release of bioactive molecules such as BMP-2 and VEGF, synergistically enhancing angiogenesis and osteogenesis. In this context, pore size, interconnectivity, mineral or ion incorporation, and growth-factor affinity directly influence vascular ingrowth, osteoblast recruitment, nutrient exchange, osteogenic differentiation, and mineralized matrix formation [303,307]. Controlled release is also critical because early angiogenic stimulation may support vascular invasion, whereas sustained osteoinductive signaling may promote later-stage bone formation and remodeling. Therefore, the release behavior of fracture-oriented hydrogels should be designed according to the temporal sequence of vascularization, osteogenesis, and mechanical maturation [307].

Particularly in the context of sports rehabilitation, protein hydrogels can dynamically respond to external mechanical stimulation, support early functional training, and accelerate the bone healing process. Nevertheless, this potential should be interpreted cautiously because pure protein hydrogels are generally insufficient as standalone load-bearing substitutes for large or mechanically demanding bone defects [34]. In sports-related fractures, especially those exposed to early rehabilitation loading, hydrogels are more realistically positioned as adjunctive matrices that support local delivery, vascularization, mineralization, and defect microenvironment regulation [299] rather than as independent replacements for internal fixation or bone grafts. Thus, protein-based hydrogels serve not only as structural fillers but also as regulators of the microenvironment and mechanical adaptation, opening new avenues for functional bone regeneration in sports-related fractures. Overall, the design of protein-based hydrogels for fracture repair should follow a structure–property–function logic, in which network stiffness, mineralization support, degradation space, angiogenic and osteogenic release, and mechanical stabilization are coordinated to promote reliable bone regeneration and functional recovery [299].

**Figure 12:**
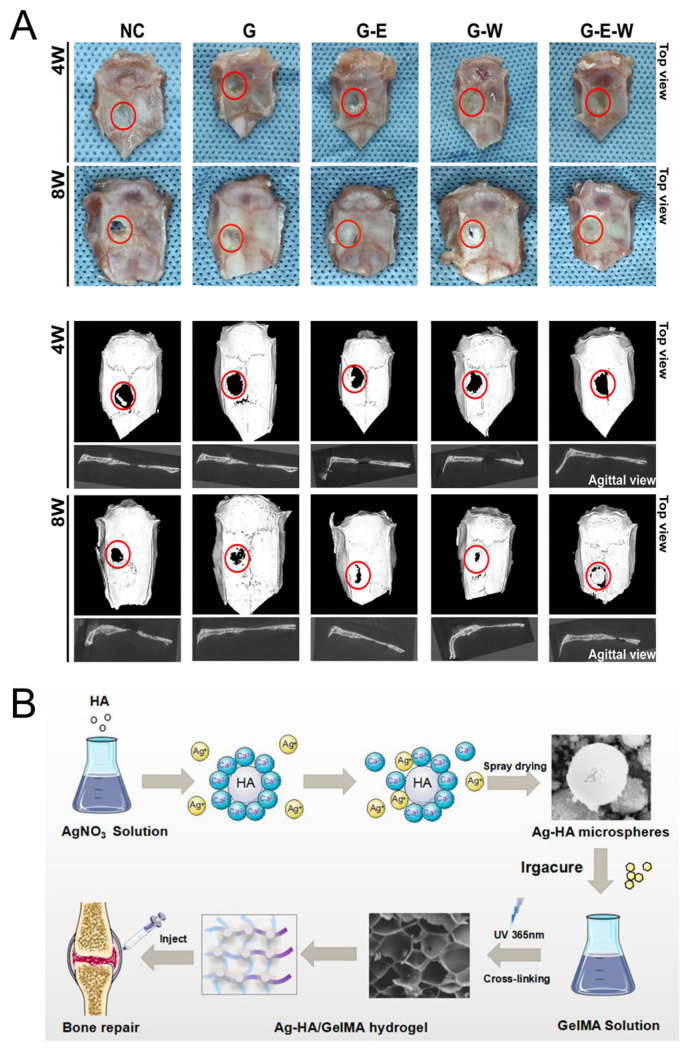
(**A**): Macroscopic and micro-CT observation of bone defect repair using the G, G-E, G-W and G-E-W artificial membranes at 4 and 8 weeks [308]. (**B**): Schematic illustration of the Ag-HA/GelMA hydrogel and the application in bone tissue engineering [312].

In promoting the proliferation of key repair cells (such as osteoblasts, chondrocytes, and MSCs), protein-based hydrogels provide multiple approaches. One strategy is to incorporate bioactive components into hydrogels to enhance cell proliferation and differentiation. For example, Zhang et al. constructed an artificial periosteum hydrogel membrane (G-E-W) composed of gelatin methacryloyl (GelMA), E7 peptide, and Wharton’s jelly-derived bioactive components. In this system, GelMA provided the structural and mechanical support of the membrane, E7 peptide endowed the membrane with BMSC-recruiting ability, and Wharton’s jelly supplied biological cues for proliferation and osteogenic differentiation. In vitro, the G-E-W membrane recruited BMSCs and promoted cell proliferation and osteogenic differentiation. In a rat skull defect model, macroscopic observation and micro-CT evaluation at 4 and 8 weeks showed that the G-E-W group was superior to the blank control and single-component membrane groups in both the quantity and quality of newly formed bone. These findings indicate that the combination of stem cell recruitment, bioactive support, and GelMA-based structural stability can effectively mimic periosteal function and promote bone defect repair (Figure 12A) [308]. In addition to growth factor incorporation, plant-derived extracts have been used to stimulate osteogenesis. Leal et al. prepared a GelMA hydrogel loaded with 5% Ximenia americana L. aqueous extract and evaluated its therapeutic effect alone or in combination with LED photobiomodulation. The in vivo experiment included 50 male Wistar rats divided into five groups: control, GelMA, GelMA + LED, GelMA/Ximenia americana L., and GelMA/Ximenia americana L. + LED. Bone repair was evaluated at 15 and 30 days using histological analysis and Raman spectroscopy. At 15 days, treated groups showed significantly increased PO_4_^3−^ deposition compared with the control group, with *p* < 0.0001 for the GelMA + LED and GelMA/Ximenia americana L. groups and *p* < 0.001 for the GelMA/Ximenia americana L. + LED and GelMA groups. At 30 days, the GelMA/Ximenia americana L. + LED group showed a significant increase in PO_4_^3−^ deposition compared with the control group (*p* < 0.0001). Histological analysis further showed that the GelMA/Ximenia americana L. and GelMA/Ximenia americana L. + LED groups presented more compact newly formed bone tissue with cortical-like maturation. These results suggest that Ximenia americana L. extract incorporated into GelMA can accelerate early bone repair, while combination with LED photobiomodulation further improves bone strengthening and maturation at later stages [309]. Beyond growth factors and plant extracts, some studies have explored dual-action strategies using drugs and metal ions. Ma developed GelMA-based microspheres (IOHM-AS-Mgs) loaded with alendronate sodium (AS) and magnesium ions (Mg^2+^). These microspheres exhibited excellent cell compatibility and adhesion, while Mg^2+^ strongly stimulated osteogenesis. In vitro, IOHM-AS-Mgs significantly enhanced osteogenic activity of BMSCs, as evidenced by the highest alkaline phosphatase (ALP) activity and mineralized nodule formation. Western blot and qPCR analyses confirmed upregulation of osteogenic markers Runx2 and BMP2, thereby promoting bone regeneration. Additionally, these hydrogels enhanced angiogenesis to support bone repair. RNA-seq results showed upregulation of osteogenic genes and suppression of inhibitory pathways, suggesting involvement of BMP/Smad signaling in MSC osteogenic differentiation [310]. Since fractures are often accompanied by open injuries and surgical interventions, the risk of infection and immune response is an important concern. The natural origin of protein-based hydrogels offers advantages of low immunogenicity, while their ability to encapsulate drugs makes them well-suited for infection prevention and immune modulation, thereby expediting fracture healing. Among antibacterial strategies, metal ions play a particularly important role. Wang developed an injectable antibacterial hydrogel (Ag-HA/GelMA), mimicking the organic–inorganic characteristics of natural bone ECM. The hydrogel combined GelMA’s biocompatibility and adhesion with the strong antibacterial properties of silver ions and the mechanical reinforcement of hydroxyapatite (HA) microspheres (Figure 12B). Mechanical testing also showed improved structural resistance, with the maximum loading force reaching 13 gf and 31 gf in the 10% and 15% GelMA groups, respectively. After Ag^+^ incorporation, Ag-HA/GelMA showed clear antibacterial activity. The inhibition zones of 1% and 3% Ag-HA/GelMA were 14.9 and 15.3 mm against Staphylococcus aureus and 15.7 and 16.1 mm against Escherichia coli, respectively. Cytocompatibility assays using MC3T3-E1 cells demonstrated continued cell proliferation over 1, 3, and 5 days, and the cell viability remained above the 70% threshold defined by ISO 10993 (Biological evaluation of medical devices—Part 1: Evaluation and testing within a risk management process) [311] for non-obvious cytotoxicity. These results suggest that Ag-HA/GelMA can provide antibacterial protection, injectable defect filling, and an osteoblast-compatible microenvironment for bone tissue engineering [312]. Similarly, Wan designed a zinc–strontium phosphate-doped galloyl-gelatin hydrogel (GGA-ZSP). Strontium ions (Sr^2+^) stimulated osteoblast and endothelial cell activity, suppressed osteoclast function, and promoted MSC osteogenic differentiation, while zinc ions (Zn^2+^) provided antibacterial activity, maintained an anti-inflammatory environment, and facilitated bone mineralization. Both Sr^2+^ and Zn^2+^ promoted angiogenesis, ensuring sufficient blood supply. In rat calvarial defect models, GGA-ZSP hydrogels exhibited excellent injectability, biocompatibility, and significantly enhanced bone regeneration, highlighting their translational potential for clinical bone repair [313].

For bone fracture repair, protein-based hydrogels are useful as carriers for osteoinductive factors, cells, ions, or mineral components, but they are generally insufficient as standalone load-bearing materials [314]. Bone healing requires mechanical stability, vascularization, mineral deposition, and remodeling under physiological loading [299,303]. Pure protein hydrogels often lack adequate stiffness, mineral conductivity, and long-term structural stability for large or load-bearing bone defects [304]. Therefore, their role may be more appropriate as adjunctive systems combined with internal fixation, inorganic fillers, bioactive ceramics, or osteogenic growth factors rather than as independent substitutes for bone grafts. Future studies should evaluate not only bone volume or histological mineralization, but also mechanical strength, vascular integration, degradation–mineralization matching, implant compatibility, infection risk, and long-term remodeling in clinically relevant defect models [304,307].

### 5.5. Limitations and Translational Challenges of Protein-Based Hydrogels

Protein-based hydrogels also face important limitations and translational challenges in sports injury repair. Although they possess favorable biocompatibility, biodegradability, and extracellular matrix-mimicking properties, their natural protein origin may introduce batch-to-batch variability, possible immunogenicity, and instability during storage or processing [315,316]. These issues are particularly important for clinical translation because variations in protein source, purification degree, molecular weight distribution, and crosslinking efficiency may directly affect hydrogel mechanics, degradation behavior, biological activity, and safety profiles [317]. Therefore, before protein-based hydrogels can be widely applied in sports medicine, their translational evaluation should include not only cytocompatibility or short-term histological repair, but also batch reproducibility, mechanical consistency, degradation stability, and strict quality-control standards [263].

Sterilization represents another critical barrier. Conventional sterilization methods, including heat treatment, gamma irradiation, ethylene oxide, and chemical sterilants, may alter protein conformation, crosslinking density, gelation behavior, swelling properties, mechanical strength, degradation rate, and bioactive molecule retention [318]. As a result, a hydrogel that performs well under laboratory conditions may lose part of its biological or mechanical function after sterilization [319]. This problem is particularly relevant for injectable, implantable, or drug-loaded hydrogel systems, where microbial safety must be achieved without compromising material performance [320]. Therefore, sterilization-related changes should be reported as part of material validation rather than treated as a technical detail [317]. Future studies should compare hydrogel properties before and after sterilization, including rheological behavior, mechanical strength, swelling ratio, degradation profile, residual toxic byproducts, release kinetics, and biological activity. Mild sterilization strategies, aseptic manufacturing processes, or lyophilized precursor systems may be useful for preserving both sterility and hydrogel functionality [321].

A further challenge is that many protein-based hydrogels still lack sufficient long-term mechanical durability for dynamic sports injury environments. Sports-injured tissues are exposed to repeated joint motion, tendon loading, muscle contraction, local inflammatory enzymes, and rehabilitation-related mechanical stress. Under these conditions, hydrogels may undergo fatigue damage, swelling-induced weakening, premature degradation, displacement, or uncontrolled release of therapeutic agents [322]. Therefore, favorable cell compatibility or short-term histological repair under controlled experimental conditions does not necessarily indicate that a hydrogel can maintain its function during rehabilitation loading. In addition, hydrogel degradation must be carefully matched with the time course of tissue repair. Rapid degradation may cause early loss of mechanical support, reduced material retention, or premature release of bioactive factors before sufficient tissue remodeling occurs. Conversely, excessively slow degradation may restrict cell infiltration, delay extracellular matrix remodeling, or induce persistent foreign-body or inflammatory responses [323]. Residual crosslinkers, photoinitiators, sterilization-induced byproducts, or degradation products may further affect local tissue reactions, especially in poorly vascularized tissues such as cartilage, tendon, and ligament.

From a translational perspective, the current level of evidence supporting protein-based hydrogels remains uneven across different injury types. Many studies report favorable cellular responses, anti-inflammatory effects, or histological repair in vitro or in small-animal models, but fewer studies provide long-term biomechanical testing, standardized functional outcomes, large-animal validation, or clinically relevant comparisons with established treatments [24]. This gap is particularly important for sports injury repair, where therapeutic success should not be defined only by defect filling or improved staining, but also by mechanical competence, load tolerance, pain relief, functional recovery, and readiness for progressive rehabilitation or return to sport. Therefore, future hydrogel studies should move beyond proof-of-concept bioactivity and include long-term fatigue testing, degradation–repair matching, release-kinetic analysis, immunological evaluation, sterilization validation, large-animal studies, and direct comparison with established clinical therapies [324]. Overall, the clinical value of protein-based hydrogels should be assessed through integrated outcome systems that combine material stability, tissue-level regeneration, biomechanical performance, biosafety, and functional recovery.

## 6. Conclusions

In summary, MSKUS and protein-based hydrogels provide complementary opportunities for improving the diagnosis, treatment, and rehabilitation monitoring of sports injuries. MSKUS enables dynamic evaluation of lesion location, tissue structure, vascular response, stiffness changes, and healing progression, thereby providing useful information for understanding the local repair status of injured tissues. Protein-based hydrogels, in turn, can provide localized therapeutic support through bioactive delivery, inflammation regulation, extracellular matrix reconstruction, mechanical reinforcement, and tissue-specific regeneration.

More importantly, the combination of these two approaches may support a more adaptive management pathway. Before treatment, MSKUS can help define lesion characteristics and guide the selection of hydrogel type, delivery route, and intervention timing. During treatment, ultrasound guidance may improve the precision of hydrogel injection and localization. After treatment, repeated MSKUS assessment may help monitor hydrogel retention or degradation, tissue remodeling, inflammatory resolution, scar formation, stiffness recovery, and functional movement. Therefore, integrating MSKUS-based evaluation with hydrogel-mediated repair may provide a promising direction for personalized and rehabilitation-oriented sports injury management. Nevertheless, the clinical translation of musculoskeletal ultrasound and protein-based hydrogels in sports medicine still faces several limitations:

First, large-scale manufacturing and quality control of protein-based hydrogels remain major challenges. Natural protein sources may show batch-to-batch variability in molecular weight, purity, bioactivity, and gelation behavior, which may influence mechanical properties, degradation profiles, release kinetics, and biological responses. Therefore, future studies should establish standardized production systems and quality-control parameters, including protein concentration, degree of modification, gelation time, swelling behavior, mechanical strength, degradation rate, release profile, residual crosslinkers, endotoxin level, sterility, and storage stability. For clinically used injectable or implantable hydrogels, sterilization methods should also be carefully optimized to maintain protein conformation, crosslinking behavior, mechanical integrity, and bioactivity.

Second, regulatory considerations and biosafety evaluation should be more clearly addressed. Depending on their composition and intended use, protein-based hydrogels may be classified as medical devices, drug delivery systems, biologics, or combination products. Hydrogels containing growth factors, cells, nanoparticles, or pharmacological agents may require more complex regulatory evaluation than simple scaffold materials. Key issues include raw material traceability, manufacturing consistency, degradation products, immunogenicity, residual monomers or photoinitiators, sterilization validation, and long-term safety monitoring. Establishing clear regulatory pathways will be essential for translating protein-based hydrogel systems from laboratory research to clinical sports medicine.

Third, cost-effectiveness should be considered when developing MSKUS–hydrogel strategies for sports injury management. MSKUS is relatively accessible, portable, and suitable for repeated follow-up, which may reduce reliance on expensive imaging modalities in selected rehabilitation scenarios. However, advanced hydrogel systems involving recombinant proteins, bioactive factors, cells, or personalized fabrication may increase treatment costs. Future studies should therefore evaluate not only biological repair efficacy, but also total healthcare cost, number of follow-up visits, imaging requirements, rehabilitation duration, return-to-sport time, recurrence rate, and patient-reported outcomes. Such analyses will help determine whether MSKUS-guided hydrogel therapy provides practical value compared with conventional treatment or non-guided delivery.

Fourth, standardized clinical workflows are needed before broad application. A feasible workflow may include patient screening, baseline MSKUS assessment, lesion localization and stratification, hydrogel indication assessment, delivery-route selection, ultrasound-guided injection or localization, immediate safety confirmation, longitudinal MSKUS monitoring, rehabilitation adjustment, and return-to-sport decision-making. In this workflow, MSKUS should not only be used for diagnosis, but also for treatment planning, delivery guidance, therapeutic-response monitoring, and rehabilitation feedback. Clear clinical criteria should be developed to determine which injury types are suitable for hydrogel-based intervention, when the material should be delivered, how the repair process should be monitored, and how imaging findings should guide progressive loading and functional recovery.

Fifth, more systematic laboratory, preclinical, and clinical studies are needed to validate the combined use of MSKUS and protein-based hydrogel therapy. In vitro and ex vivo experiments should first be performed to evaluate hydrogel injectability, ultrasound visibility, localization stability, degradation behavior, mechanical evolution, and release kinetics under tissue-like conditions. Animal models of muscle, tendon, cartilage, osteochondral, and tendon–bone injuries should then be used to assess hydrogel retention, inflammatory regulation, vascular response, matrix remodeling, stiffness recovery, and functional repair under repeated MSKUS monitoring. On this basis, well-designed clinical trials should compare MSKUS-guided hydrogel intervention with conventional treatment or non-guided delivery, using standardized endpoints such as pain scores, functional scales, imaging-based healing parameters, hydrogel retention or degradation, return-to-sport time, and adverse events. These stepwise studies will be essential for determining whether MSKUS-guided protein-based hydrogel therapy can be translated into practical sports injury management.

Overall, musculoskeletal ultrasound and protein-based hydrogels hold considerable promise for sports injury management by linking dynamic lesion assessment with localized biomaterial-assisted repair. In this integrated strategy, MSKUS can help characterize lesion features before intervention, support more precise hydrogel delivery when appropriate, and provide repeated information on local tissue response during follow-up. These imaging findings may further assist in adjusting rehabilitation intensity and evaluating readiness for progressive loading or return to sport. However, successful translation will depend not only on imaging accuracy and biomaterial performance, but also on scalable manufacturing, regulatory validation, cost-effectiveness, standardized clinical workflows, material-specific monitoring strategies, and rigorous experimental and clinical evidence. Future studies should further validate MSKUS-guided protein-based hydrogel strategies through standardized imaging protocols, functional outcome measures, long-term safety assessment, and clinically relevant comparative studies. With continued advances in imaging systems, biomaterials science, and rehabilitation medicine, this integrated approach may provide a more precise and individualized strategy for sports injury diagnosis, treatment, and functional recovery.

## Figures and Tables

**Table 1:** Overview of major sports injuries and treatments.

Injury Type	Cause of Injury	High-Risk Population	Method of Treatment	Preventive Measures	Refs.
Cartilage Injury	Mechanical imbalance; Inflammatory cytokines; Aging-related chondrocyte apoptosis; Traumatic impact	Basketball/football athletes; Osteoarthritis patients > 45 years; Obese individuals; Meniscus tear patients	Symptomatic management; Arthroscopic lavage; MACI; Joint replacement for severe lesions	Maintain joint alignment; Weight control; Avoid high-impact loading; Regular evaluation	[16,17,18,19,20,21,22,23]
Muscle Injury	Acute strain; Chronic overload; Metabolic disorders; Indirect trauma	Sprinters and football players; Fitness enthusiasts; Middle-aged and elderly individuals; Manual laborers	RICE principle; NSAIDs (e.g., ibuprofen) for pain and inflammation; Progressive resistance training to restore muscle strength; Physical therapy to promote blood flow and repair	Adequate warm-up; Proper movement technique; Rational training plans; Regular rest for occupational workers	[24,25,26,27,28,29,30]
Tendon Injury	Mechanical overload; Technical errors; Degenerative tendinopathy; Inadequate equipment	Tennis/badminton players; Ball-sport athletes aged 30–50; Factory workers; Novice gym trainees	Eccentric training; Extracorporeal shockwave therapy; Corticosteroid injection; Open surgical repair for severe rupture	Correct movement technique; Proper footwear and equipment; Regular tendon stretching for repetitive tasks	[31,32,33,34,35,36,37]
Bone Fracture	High-energy trauma; Osteoporotic bone loss; Stress microfracture accumulation; Pathological destruction	Young athletes; Older adults; Postmenopausal women; Professional athletes	Internal fixation; External fixation for open fractures; Cast or brace immobilization; Calcium + Vitamin D supplementation	Calcium and vitamin D supplementation; Fall prevention; Proper protection during sports	[38,39,40,41,42,43,44]

**Table 2:** Proposed closed-loop workflow for MSKUS-guided protein-based hydrogel therapy in sports injuries.

Workflow Stage	Main Purpose	Role of MSKUS	Hydrogel-Related Decision	Follow-Up or Decision Indicator	Refs.
Pre-intervention assessment	Define lesion phenotype	Evaluate injury type, depth, structural disruption, vascularity, effusion, and stiffness	Determine whether hydrogel therapy is suitable	Lesion accessibility, defect size, hematoma, effusion, stiffness	[160,161,162,163]
Lesion stratification	Match pathology with material function	Distinguish tear, degeneration, inflammation, effusion, or interface injury	Select anti-inflammatory, regenerative, adhesive, lubricating, or mechanically supportive hydrogel	Tissue type, inflammatory activity, mechanical demand	[21,164,165,166]
Delivery planning	Identify safe and effective route	Plan needle path and avoid vessels, nerves, and intact load-bearing structures	Choose injectable, adhesive, intra-articular, or defect-adjacent delivery	Needle trajectory, tissue depth, target region	[167,168,169,170]
Ultrasound-guided administration	Localize material placement	Track needle tip and hydrogel distribution in real time	Adjust injection site, dose, and distribution	Target coverage, leakage, non-target diffusion	[142,170,171,172,173]
Immediate confirmation	Verify placement and early safety	Confirm localization and detect hematoma, displacement, or leakage	Decide whether additional delivery or repositioning is needed	Retention, distribution, early complication	[155,174,175,176]
Longitudinal monitoring	Assess material behavior and tissue response	Monitor volume, boundary, swelling, Doppler signal, tissue continuity, stiffness, and effusion	Adjust follow-up schedule and rehabilitation loading	Retention/degradation, inflammation, matrix remodeling	[177,178,179,180]
Rehabilitation feedback	Guide functional recovery	Evaluate dynamic movement, tendon gliding, muscle contraction, and joint effusion under loading	Inform progressive exercise and return-to-sport decision	Functional readiness, symptom recurrence, structural stability	[161,163,177,178]

**Table 3:** Overview of protein-based hydrogel strategies for different sports injury repair scenarios.

Type of Sports Injury	Advantages of Protein-Based Hydrogels	Therapeutic Mechanisms	Application Approaches	Refs.
Muscle Injury	Excellent biocompatibility for satellite cell activation; ECM-mimicking 3D porous architecture guiding myoblast alignment; High plasticity ensuring defect conformity and mechanical stability; Tunable degradation synchronized with muscle regeneration	Myogenesis promotion—Sustained release of myogenic factors (IGF-1, HGF) activates satellite cells; Defect filling—Sol–gel transition enables in situ filling and scar prevention; Scar inhibition—Degradation byproducts suppress fibroblast overactivation; Fiber alignment—Fibrillar structure guides myofiber orientation; Anti-inflammatory modulation—IL-10/TGF-β3 reduce M1 macrophage polarization	Wound coverage: Hydrogel membranes act as hemostatic and protective dressings for open injuries; Injectable application: Minimally invasive injection of sol-state hydrogels that undergo in situ gelation; Stem cell co-injection to enhance cell engraftment and regeneration	[206,207,208,209,210,211]
Cartilage Injury	Hydrophilic porous structure supporting cell viability; Tunable elasticity matching native cartilage; Efficient carrier for targeted cell delivery; Biocompatible matrix maintaining chondrocyte phenotype	Nutrient and ECM support—Porous network enhances nutrient diffusion and ECM exchange; Matrix synthesis stimulation—TGF-β3/IGF-1 promote collagen II and proteoglycan deposition; Mechanical compatibility—Tunable crosslinking matches cartilage compressive modulus; Phenotype preservation—ECM-mimetic structure prevents chondrocyte dedifferentiation; Targeted cell delivery—Encapsulated chondrocytes/MSCs enable localized repair and integration	Intra-articular injection: Direct injection into cartilage defects (e.g., knee lesions); Post-microfracture application leveraging enhanced vascular access; Layered implantation: Gradient-pore hydrogels reconstruct osteochondral interfaces for deep cartilage repair	[212,213,214,215,216,217,218]
Ligament Injury	Fibrous structure mimicking native collagen bundles; Elastic and tough matrix enhancing tensile strength; Anti-adhesion barrier improving joint mobility	Ligament regeneration—PDGF-BB-loaded hydrogels promote fibroblast proliferation and collagen synthesis; Mechanical restoration—Tunable crosslinking and fiber alignment match native ligament modulus; Anti-adhesion barrier—Prevents postoperative connective tissue attachment; Antioxidant defense—GSH/Vitamin E reduce ROS and oxidative stress; Structural reinforcement—Hydrogel encapsulation at suture sites improves tensile strength and prevents reinjury	Intra-articular injection: Arthroscopic injection into partial ligament tears (e.g., ACL); Post-reconstruction injection to prevent adhesions; Scaffold implantation: Preformed fibrous hydrogels implanted into ligament gaps to guide axially aligned growth	[219,220,221,222,223,224]
Bone Fracture	Carrier for osteoinductive factors enhancing osteogenesis; Adhesive matrix stabilizing fracture alignment; Nanocomposite promoting mineralization; Biodegradable scaffold avoiding secondary surgery	Callus formation—BMP-2/VEGF co-delivery induces coupled osteogenesis and angiogenesis; Accelerated mineralization—Hydroxyapatite/TCP enhance calcium deposition and matrix hardening; Fracture stabilization—Adhesive hydrogel maintains alignment and limits micromotion; Anti-inflammatory regulation—NSAID-loaded hydrogels reduce IL-6/TNF-α expression and swelling	Combined with internal fixation: Intraoperative filling around plates or screws; Coating modification of implants for enhanced osseointegration; Injectable delivery: Percutaneous injection for closed fractures; Vertebroplasty adjunct for osteoporotic compression fractures	[225,226,227,228,229]

## Data Availability

Not applicable.
